# A Programmable Calcification Nanoplatform for Loco‐Regional Calcification‐Immune Hepatocellular Carcinoma Therapy

**DOI:** 10.1002/advs.76642

**Published:** 2026-07-20

**Authors:** Long Liu, Peng Li, Zhixiang Lu, Shuqin Xu, Juanjuan Wang, Kai Ma, Haodong Yu, Can Zhou, Hu Chen, Gang Liu, Yi Lyu, Shuang Bai

**Affiliations:** ^1^ Shaanxi Province Center for Regenerative Medicine and Surgery Engineering Research The First Affiliated Hospital of Xi'an Jiaotong University Xi'an China; ^2^ National Local Joint Engineering Research Center for Precision Surgery & Regenerative Medicine The First Affiliated Hospital of Xi'an Jiaotong University Xi'an China; ^3^ Department of Hepatobiliary Surgery The First Affiliated Hospital of Xi'an Jiaotong University Xi'an China; ^4^ Institute of Regenerative and Reconstructive Medicine Med‐X Institute The First Affiliated Hospital of Xi'an Jiaotong University Xi'an China; ^5^ State Key Laboratory of Cellular Stress Biology Innovation Center for Cell Biology School of Life Sciences Xiamen University Xiamen China; ^6^ Department of Breast Surgery The First Affiliated Hospital of Xi'an Jiaotong University Xi'an China

**Keywords:** calcium overload, in situ growth synthesis, MCOLN2, mitochondrial dysfunction, tumor calcification

## Abstract

Inducing tumor calcification is a promising strategy for disrupting cancer progression, yet achieving efficient biomineralization with synergistic antitumor effects remains challenging. Here, a programmable calcification nanoplatform (CaIM) is developed using in situ growth of calcium peroxide (CaO_2_) on black phosphorus (BP) nanosheets, followed by hyaluronic acid encapsulation. Unlike conventional physical adsorption or co‐precipitation, this in situ growth strategy yields crystalline CaO_2_ uniformly anchored on the BP matrix, enabling synchronized release of Ca^2^^+^, phosphate, and H_2_O_2_ in the tumor microenvironment. CaIM induces potent intracellular calcium overload and oxidative stress, triggering mitochondrial dysfunction, cell death, and extensive biological calcification readily visualized via computed tomography (CT). Multi‐omics profiling identifies the mucolipin channel MCOLN2 as a key mediator regulating calcium flux, facilitating stable active biomineralization rather than passive necrotic calcification. This active calcification, coupled with mitochondrial damage, effectively reshapes the immunosuppressive microenvironment by promoting macrophage polarization and T cell infiltration. These findings establish a calcium signaling‐driven paradigm integrating nanocomposite design with synergistic calcium stress, oxidative damage, and immune activation, offering a robust foundation for calcification‐based cancer therapy.

## Graphical abstract

An in situ‐grown BP‐CaO_2_ nanoplatform supplies coordinated Ca^2^^+^, endogenous phosphate, and oxidative stress to convert tumor calcification from a passive endpoint into an active immune‐remodeling process. Widespread hydroxyapatite deposition is visualized by CT, while multi‐omics reveals MCOLN2 as a calcium‐responsive mediator linking biomineralization to M1 polarization and therapeutic outcome.

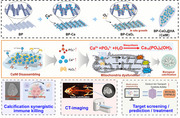

## 1 Introduction

Cancer research progresses from clinical observations to mechanistic understanding, which in turn lays the foundation for precision therapy. Recently, tumor calcification as a phenomenon frequently detected in computed tomography (CT) scans following radiotherapy or chemotherapy has emerged as a valuable marker for evaluating therapeutic efficacy [1, 2]. Clinical and basic investigations, particularly in hepatocellular carcinoma (HCC), have demonstrated that the occurrence of intratumoral calcification post‐treatment is strongly correlated with higher complete response rates and longer progression‐free survival [3, 4, 5]. Tumor calcification occurred in 38% after radioembolization treatment in HCC, exhibiting a 71.4% complete response and markedly improved 1 and 2‐year survival (94.7% and 86.1%) compared with non‐calcified tumors [3]. Growing evidence has inspired increasing interest in exploring calcification as a proactive and therapeutically inducible paradigm, rather than merely a passive consequence of therapy, offering new avenues for tumor control through engineered intervention promoting biomineralization.

Calcification represents a pathological process, driven by local supersaturation of mineral ions such as calcium ions (Ca^2^^+^), phosphate ions (PO_4_^3^^−^), carbonate ions (CO_3_^2^^−^), etc. Mobile ions first arrive at diseased tissue, reach local supersaturation, pair with counter‐ions, and ultimately precipitate as a solid (e.g., hydroxyapatite), thereby reshaping tissue mechanics and disrupting cellular homeostasis [6]. Moreover, tumor calcification extends beyond physicochemical ion supersaturation; it can be an active biological response regulated by the cellular redox status and ion signaling, and this promoting effect reduces the energy barrier for mineral nucleation. Emerging evidence suggests that tumor biological calcification could exert broader effects on cellular homeostasis, potentially affecting mitochondrial metabolism, redox balance, and immune regulation [7, 8, 9]. Perturbation of calcium homeostasis and induction of calcium overload can disrupt mitochondrial integrity, amplify reactive oxygen species (ROS) generation, and trigger cell death [7, 10]. Meanwhile, the elevated intracellular Ca^2^^+^ levels have been implicated in remodeling the tumor immune microenvironment (TME) and promoting immunotherapy [11]. Controlled modulation of calcium dynamics may offer a promising approach to synergistically link Ca^2^^+^ overload with tumor calcification and immune remodeling, accelerating the clinical translation of tumor calcification therapy.

Additionally, tumor calcification is not merely a static radiological feature, involving a complex, intricate interplay, such as calcium signaling, oxidative stress, biomineralization, and immune activation [12]. Traditional evaluation of tumor calcification relies predominantly on imaging techniques such as CT, which can visualize mineral deposition but fails to capture the underlying molecular events driving this process. Owing to the fact that the molecules linking these processes remain largely undefined, seeking specific molecular biomarkers is valuable that can indicate tumor calcification and predict patient prognosis. Multi‐omics technologies provide an integrated framework to capture the complexity of calcium signaling and biomineralization at multiple biological levels. By jointly analyzing protein abundance, phosphorylation dynamics, gene expression, and cellular heterogeneity, multi‐omics profiling enables the systematic identification of candidate biomarkers and signaling pathways associated with tumor calcification. Such approaches could establish a foundation for discovering predictive molecular signatures [13, 14], which may complement imaging assessment and guide clinical prognosis in tumor calcification therapy.

**FIGURE 1:**
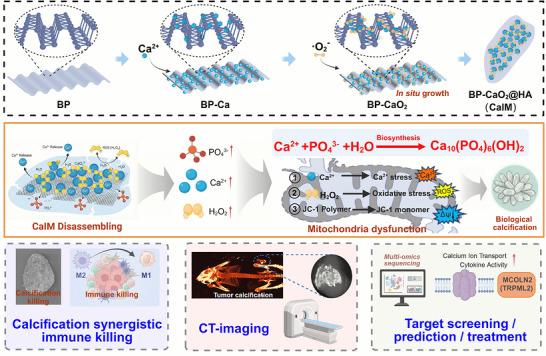
Schematic illustration of in situ growth synthesis of BP‐CaO_2_ and the resulting mediation of diagnosis, therapy, and their mechanisms. CaO_2_ is synthesized on black phosphorus (BP) nanosheets via in situ growth (top), yielding BP‐CaO_2_ nanoparticles that are subsequently encapsulated with hyaluronic acid to form BP‐CaO_2_@HA. After endocytic uptake, CaIM disassembles in the tumor microenvironment and releases Ca^2^^+^, PO_4_^3^^−^, and H_2_O_2_. Excess Ca^2^^+^, PO_4_^3^^−^, and oxidative stress drive biological calcification, while calcium stress, oxidative stress, and the decrease in mitochondrial membrane potential all jointly led to mitochondrial dysfunction, which further promoted the related cell death and tumor calcification. Calcium signaling further recruits and polarizes macrophages toward the M1 phenotype, reshaping the tumor microenvironment and eliciting robust anti‐tumor immunity. Furthermore, the calcification, with its CT imaging characteristics, can be used for tumor diagnosis. Multi‐omics profiling identifies MCOLN2 as a calcium‐responsive biomarker linked to immune activation and calcification. Together, these effects suppress tumor growth and enable CT‐based imaging of intratumoral calcification, calcification‐induced synergistic immune killing, and biomarker‐guided prediction and treatment.

Herein, we innovatively construct a calcification immunology modulator (CaIM) through in situ growth synthesis of CaO_2_ on black phosphorus (BP) nanosheets, encapsulated within hyaluronic acid. BP, as a two‐dimensional layered material with high carrier mobility and intrinsic biodegradability, provides an ideal carrier. The expansive specific surface area of the BP matrix accommodates substantial CaO_2_ loading, whereas the reductive microenvironment established by phosphorus lone‐pair electrons effectively prevents premature peroxide decomposition [15]. Crucially, the metabolic susceptibility of BP serves a dual purpose, including yielding a sustained flux of phosphate ions and providing the indispensable precursors for in situ hydroxyapatite nucleation. Such an integrated design provides a robust molecular framework for transitioning from passive calcification to a controllable, therapeutic biomineralization process. In terms of therapeutic function, this in situ growth‐derived nanocomposite enables synchronized calcium overload and phosphate supply within the tumor microenvironment, thereby achieving tumor calcification and immune remodeling for potent antitumor efficacy. CaIM exerts therapeutic efficacy through a coordinated mechanism, inducing calcium overload, initiating localized calcium phosphate deposition, and triggering oxidative stress, mitochondrial dysfunction, immune activation, and cell apoptosis (Figure 1). Moreover, the mucolipin channel MCOLN2 was identified as a calcium‐responsive molecular mediator that responds to CaIM‐induced ion perturbations. MCOLN2‐mediated signaling not only accelerates the kinetics of voluntary biomineralization but also drives macrophage polarization from the M2 to M1 phenotype, thereby reversing immune suppression. By integrating MCOLN2 as a molecular signature with CT imaging, we provide a holistic therapeutic evaluation strategy. Clinically, there is a large and underserved population for whom current options lack both durable immune activation and a quantifiable imaging endpoint for response assessment. The CaIM bridges a critical gap between existing loco‐regional therapies and the emerging paradigm of calcification‐based immune oncology. Overall, we synergized calcium signal with endogenous biological responses, paving the way for the clinical translation of active biomineralization therapy.

## 2 Results and Discussion

### 2.1 Synthesis and Characterization of BP‐CaO_2_ Nanostructures

**FIGURE 2:**
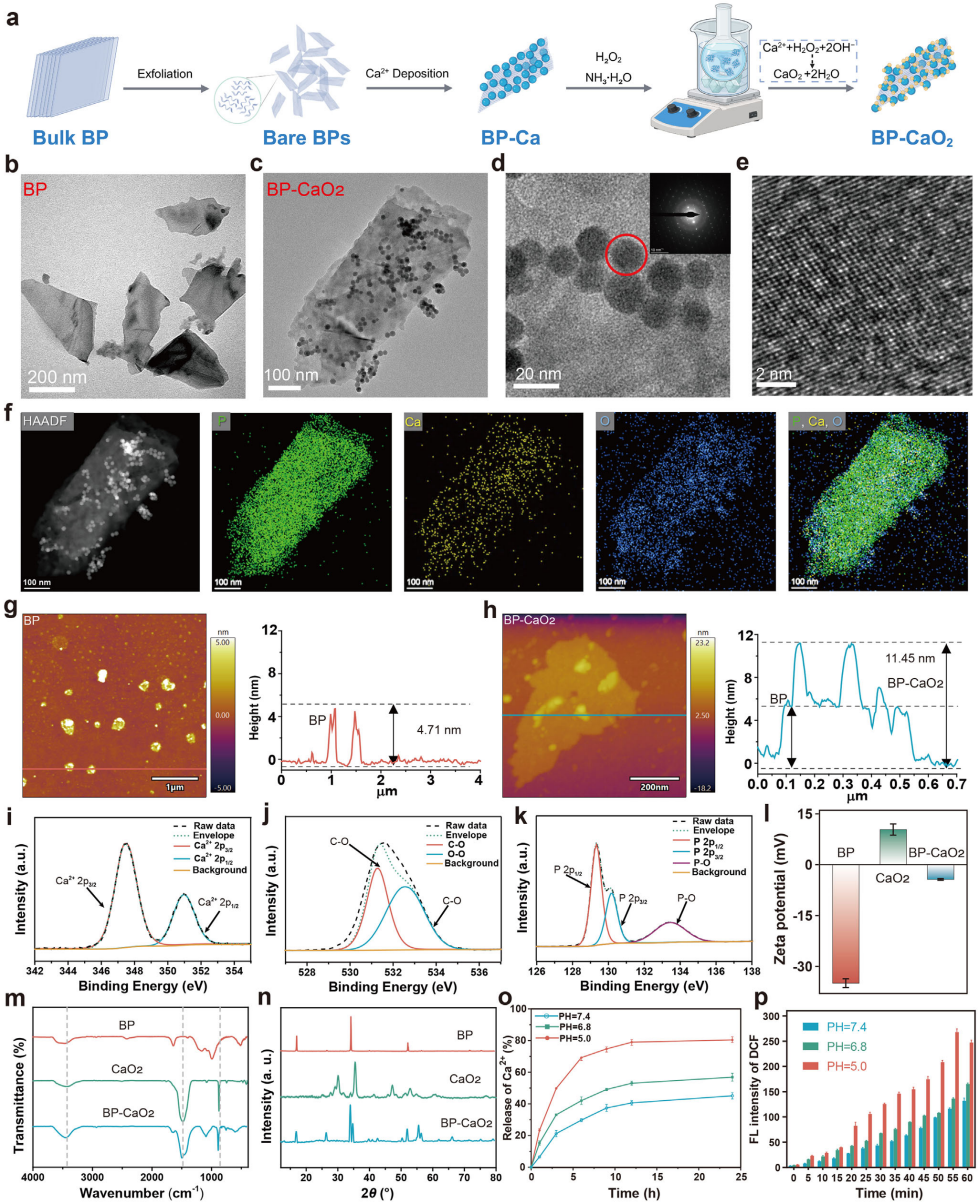
Design strategy and material characterization of BP‐CaO_2_. (a) Schematic illustration of the preparation procedure. (b) Transmission electron microscopy (TEM) images of the pristine sheets, BP. (c) TEM images of BP‐CaO_2_. (d, e) High‐resolution transmission electron microscopy (HRTEM) and corresponding lattice fringes on the BP‐CaO_2_. (f) HAADF‐STEM image and element mapping of P, Ca, and O. (g, h) AFM topography and height profiles comparing the BP nanosheets and the BP‐CaO. (i–k) High‐resolution XPS spectra of Ca 2p (i), O 1s (j), and P 2p (k) collected from the three formulations for surface chemical analysis. (l) Zeta potential of BP, CaO_2_, and BP‐CaO_2_. (m) FTIR spectra collected from BP, CaO_2_, and BP‐CaO_2_. (n) XRD patterns of BP, CaO_2_, and BP‐CaO_2_. (o) Time‐dependent Ca^2^^+^ release profiles under different pH conditions. (p) Production of H_2_O_2_ was measured at various pH environments.

Numerous studies have reported the preparation of two‐dimensional drug composites through physical adsorption, co‐encapsulation, co‐precipitation, and other methods, but the approach of in situ growth of peroxides on the surface of two‐dimensional (2D) materials and their corresponding structures has rarely been reported. Here, we introduced an innovative in situ growth methodology for the synthesis of BP‐CaO_2_ and conducted comprehensive structural characterization. The synthesis schematic diagram from BP to BP‐CaO_2_ was illustrated in Figure 2a. First, BP nanosheets were obtained by ultrasonication‐assisted liquid exfoliation for at least 12 h under low‐temperature and dark conditions. After low‐speed centrifugation to remove large precipitates, the resulting supernatant comprises single‐layer BP nanosheets. Transmission electron microscope (TEM) image indicated the exfoliated BP possesses a smooth and layered sheet‐like morphology (Figure 2b). Subsequently, BP nanosheets were mildly oxidized in an ammonia‐containing NMP solution to activate ionic binding sites. Calcium chloride was then added to trigger in situ calcium ion deposition, yielding the BP‐Ca intermediate. The final BP‐CaO_2_ was synthesized via in situ growth of CaO_2_ by introducing H_2_O_2_ and NH_3_·H_2_O to the BP‐Ca solution. The BP‐CaO_2_ prepared by in situ growth has a significantly different surface from the traditional adsorption/deposition/coating methods. TEM revealed that BP‐CaO_2_ exhibits a unique hierarchical nanostructure composed of a 2D BP substrate with 3D spherical particles (Diameter: ∼14 nm) uniformly immobilized on its surface. During the reaction, through optimizing the reaction to proceed in a water‐methanol binary solution and precisely controlling the precursor concentration and reaction temperature, we prevented the uncontrolled rapid growth of calcium peroxide, thereby ensuring a narrow size distribution of the spherical particles and their close arrangement to form the three‐dimensional heterogeneous structure shown in Figure 2c.

High‐resolution transmission electron microscopy (HRTEM) was employed to elucidate the crystallographic features of the in situ grown CaO_2_ on BP nanosheets. As shown in Figure 2d, the surface‐decorated nanoparticles exhibited pronounced crystalline contrast, indicating the formation of ordered nanodomains rather than amorphous deposits. The corresponding fast Fourier transform (FFT) pattern displayed distinct diffraction spots accompanied by weak diffraction rings, suggesting the coexistence of preferentially oriented crystalline domains and polycrystalline characteristics, thereby confirming the high crystallinity of the CaO_2_ nanoparticles generated via the in situ growth strategy (Figure 2d). Further insight into the local atomic arrangement was obtained from an enlarged HRTEM image (Figure 2e), which resolved clear and continuous lattice fringes within the nanoparticle regions, indicative of highly ordered atomic packing. Notably, these lattice fringes were spatially confined to the nanoparticle domains and were absent in the underlying BP substrate, demonstrating that the observed periodicity originates from crystalline CaO_2_ rather than the BP support. High‐angle annular dark‐field scanning TEM (HAADF‐STEM) and the corresponding elemental mapping analysis further revealed discretely and uniform distribution of Ca and O signals on the BP surface (Figure 2f). Collectively, CaO_2_ was constructed as crystalline nanoparticles uniformly anchored on BP nanosheets through in situ growth, a structural feature that was distinct from conventional adsorption or coating composites, which often suffer from limited crystallinity or amorphous surface layers. Atomic force microscope (AFM) measurements demonstrated an increase in thickness from pristine BP (4.71 nm) to BP‐CaO_2_ (11.45 nm), further validating successful surface loading (Figure 2g,h).

To elucidate the elemental composition and chemical states of BP‐CaO_2_, x‐ray photoelectron spectroscopy (XPS) was performed. The XPS survey spectrum displayed characteristic signals of P, Ca, O, and C across the full binding energy range, confirming the successful incorporation of calcium onto the BP framework (Figure S1). High‐resolution Ca 2p spectra exhibited a well‐resolved doublet with peak maxima around 347.5 and 351 eV, corresponding to the Ca 2p_3_/_2_ and Ca 2p_1_/_2_ components, respectively (Figure 2i). The observed spin‐orbit splitting was consistent with Ca^2^^+^ species reported for calcium peroxide materials, supporting the presence of CaO2‐derived domains. The O 1s spectrum can be deconvoluted into multiple components. The blue component, centered around 531 eV, can be assigned to peroxide‐related oxygen species (O─O / O_2_^2^^−^), whereas the red component distributed in the 532–534 eV range was attributable to C─O related oxygen species, with possible contributions from surface hydroxyl or adsorbed oxygen (Figure 2j). These features indicated the coexistence of peroxide‐related oxygen species and interfacial oxygen‐containing environments in BP‐CaO_2_. The high‐resolution P 2p spectrum retained a dominant doublet around 128–131 eV, characteristic of P─P bonds in crystalline BP, together with a weaker component in the 132–135 eV region assigned to oxidized phosphorus species (P─O) (Figure 2k). This suggested that the BP lattice remains largely preserved during CaO_2_ deposition, accompanied by limited surface oxidation or coordination with oxygen‐containing species. Zeta potential analysis suggested that the surface charge shifted from negative to more positive values after CaO_2_ loading, reflecting charge neutralization by CaO_2_ (Figure 2l). Moreover, the Fourier transform infrared (FTIR) spectroscopy further supported the formation of BP‐CaO_2_, presenting characteristic absorption features in the 850–900 cm^−^^1^ region attributable to peroxide‐related O─O vibrations, as well as bands in the 1000–1100 cm^−^^1^ range associated with P─O or P═O stretching modes. Additional low‐wavenumber features are consistent with Ca─O‐related vibrational modes (Figure 2m). Powder x‐ray diffraction (XRD) patterns revealed the coexistence of diffraction peaks characteristic of BP and calcium peroxide. The main reflections of BP were retained, while additional diffraction features attributable to CaO_2_ appear in BP‐CaO_2_, without detectable impurity phases (Figure 2n). Therefore, CaO_2_ was stably anchored onto BP with well‐defined chemical states and phase composition, resulting in a structurally integrated BP‐CaO_2_ nanocomposite suitable for further functional investigations.

### 2.2 In Vitro Cytotoxicity Mechanism

Considering the stability and biocompatibility of the therapy system, hyaluronic acid (HA) was chosen as the carrier to encapsulate BP‐CaO_2_ in subsequent experiments, yielding BP‐CaO_2_@HA (CaIM) (Figure S2). As pH decreases, HA will undergo protonation and structural swelling, which promote the release of cargos. Additionally, the acidic tumor environment also accelerated the decomposition of CaO_2_. As shown in Figure 2o, CaIM exhibited a pronounced pH‐responsive Ca^2^^+^ release behavior, with significantly accelerated release under simulated acidic tumor conditions. Consistently, H_2_O_2_ generation increased markedly at lower pH values due to enhanced decomposition of CaO_2_ (Figure 2p).

**FIGURE 3:**
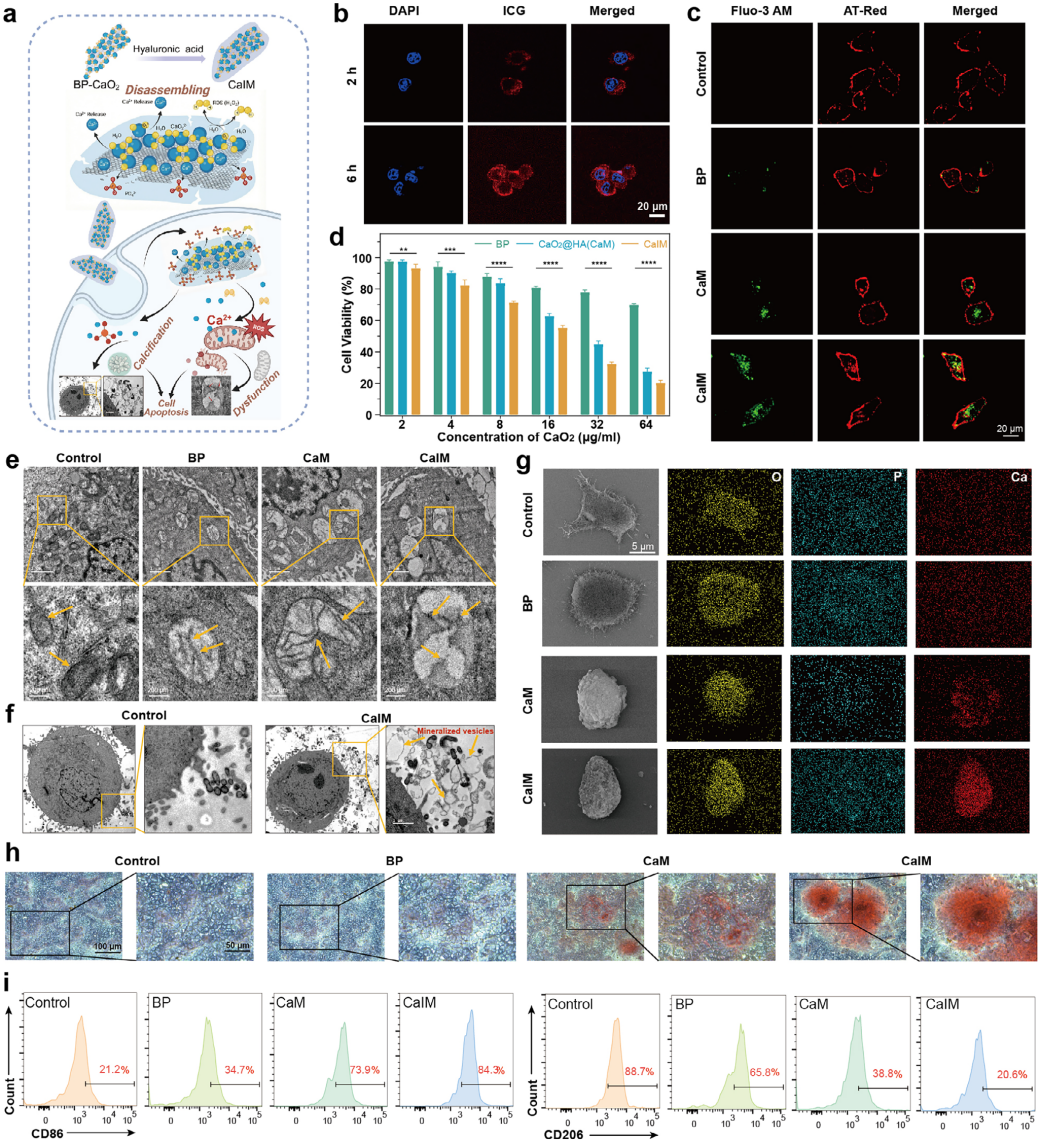
The performance of CaIM in vitro. (a) CaIM was efficiently internalized by tumor cells via endocytosis and subsequent release of Ca^2^^+^ and H_2_O_2_, resulting in intracellular calcium overload and oxidative stress. The synergistic increase in Ca^2^^+^ flux and reactive oxygen species disrupted mitochondrial homeostasis, thereby inducing mitochondrial dysfunction and triggering apoptosis‐related cellular responses. In parallel, the elevated intracellular Ca^2^^+^ and PO_4_^3−^ promoted localized calcium phosphate deposition, ultimately driving tumor calcification and contributing to tumor cell death. (b) Confocal fluorescence images exhibiting the intracellular distribution of CaIM at different incubation times. (c) Fluo‐3 AM staining for intracellular Ca^2^^+^ visualization. (d) Cell viability of tumor cells treated with the indicated formulations at various concentrations. (e) Biology transmission electron microscopy (Bio‐TEM) images of mitochondria. (f) Bio‐TEM images of intracellular vesicles observed in Control and CaIM groups. Left panel: original image; Right panel: enlarged images. (g) SEM images and corresponding EDS elemental mapping of O, P, and Ca on tumor cells. (h) Alizarin Red staining displaying calcified deposits in cells. (i) Flow cytometry analysis of immune infiltration using macrophage markers CD86 and CD206 after exposure to different formulations. ^**^ *p* < 0.01; ^***^ *p* < 0.001; ^****^ *p* < 0.0001, *n* = 3.

The cytotoxicity mechanism of CaIM was systematically investigated through the in vitro intracellular behavior and biological effects (Figure 3a). Upon cellular internalization, CaIM undergoes intracellular disassembly driven by the acidic endolysosomal environment and lysosomal hyaluronidase‐mediated HA degradation, collectively promoting cargo release and initiating the downstream therapeutic cascade. We first assessed their cell uptake behavior by flow cytometry (FCM) and confocal laser scanning microcopy (CLSM). The fluorescent dye Indocyanine Green (ICG) was loaded onto CaIM as an indicator. As shown in Figure S3, CaIM exhibited a notably time‐dependent uptake behavior, with its endocytosis rate reaching 95.9% after 6 h of co‐incubation. Images of the cells after 2 and 6 h incubation were captured using confocal microscopy. There was a distinct red fluorescence signal within the cells, indicating that CaIM had been successfully taken up by the cells (Figure 3b). Such highly efficient, time‐dependent cellular internalization provided a solid foundation for the subsequent intracellular activation and biological effects of CaIM.

To elucidate the origin of anti‐tumor cytotoxic effects, the intracellular fate of CaIM was investigated. Previous studies have shown that two‐dimensional nanosheets typically enter cells via endocytosis and progress sequentially through early endosomes, late endosomes, and lysosomes [16]. Consistent with this paradigm, lysosomal co‐localization was clearly observed at 3 h, confirming that CaIM initially accumulates in acidic lysosomal compartments (Figure S4). Such acidic environments were favorable for CaO_2_ decomposition, which released H_2_O_2_ and Ca^2^^+^, thereby priming lysosomal escape and downstream cell death pathways. Mitochondrial co‐localization experiments further revealed that a portion of CaIM reached the cytosol and interacted with mitochondria after lysosomal escape (Figure S5). This distribution enabled the direct coupling of Ca^2^^+^ overload and oxidative stress with mitochondrial vulnerability, integrating mitochondria into the attacking organelles. Meanwhile, the cellular‐level generation of ROS and Ca^2+^ from CaIM was monitored by CLSM. The control and BP group exhibited weak green fluorescence, while the group incubated with CaM and CaIM exhibited strong intracellular fluorescence, demonstrating a substantial rise in intracellular ROS levels (Figure S6). Fluo‐3 AM staining suggested that cells exposed to CaIM harbored an elevation of cytosolic Ca^2^^+^, indicating degradation and intracellular calcium overload (Figure 3c). Under physiological conditions, cytosolic free Ca^2^^+^ is tightly maintained at approximately 100–200 nm [17, 18]. Sustained elevation of cytosolic Ca^2^^+^ above 500 nm‐1 µm constitutes a well‐established critical threshold for triggering mitochondrial permeability transition pore (mPTP) opening, cytochrome c release, and activation of the caspase‐dependent intrinsic apoptotic pathway [19, 20]. The pronounced intracellular Ca^2^^+^ elevation induced by CaIM substantially exceeded this physiological range, thereby driving mPTP opening and the collapse of mitochondrial membrane potential, as confirmed by JC‐1 staining (Figure S9). The primary mode of CaIM‐induced cell death is intrinsic mitochondria‐mediated apoptosis, supported by flow cytometric annexin V/PI analysis showing marked elevation of both early and late apoptotic populations (Figure S7), and Bio‐TEM revealing characteristically swollen mitochondria with severely disrupted cristae (Figure 3e). After cellular internalization, CaIM underwent intracellular decomposition driven by the acidic environment and hyaluronidase activity, leading to the pronounced generation of Ca^2^^+^ and H_2_O_2_. The concurrent elevation of intracellular Ca^2^^+^ and H_2_O_2_ established a synergistic stress condition.

Calcium signals serve as indispensable messengers in intracellular signal transduction that orchestrate a broad spectrum of physiological processes. These processes encompass the fine‐tuned regulation of protein activity, metabolic pathways, cellular homeostasis, cell cycle progression, apoptotic programs, and cell survival mechanisms. The functional integrity of calcium signaling is critically dependent on the precise maintenance of calcium concentration gradients across cellular compartments. Any aberrant alterations in intracellular calcium levels, stemming from non‐autonomous regulatory mechanisms, have the potential to disrupt the balance of calcium signaling networks. Such disruptions can cascade into perturbations of cellular physiology, affecting multiple facets of cell function and viability. Notably, under pathological conditions characterized by oxidative stress, ROS‐mediated oxidation of thiol groups on channel proteins renders calcium channels susceptible to dysfunction, impairing the canonical regulation of intracellular Ca^2^^+^ homeostasis [21]. The resultant inability to effectively buffer intracellular Ca^2^^+^ through canonical channel pathways exacerbates calcium overload [18]. This pathological state of Ca^2^^+^ dysregulation ultimately culminates in the induction of cell death pathways, including intrinsic apoptosis mediated by mPTP opening and caspase cascade activation. To evaluate the biological effects of elevated oxidative stress and calcium signaling, cell viability assays were conducted, which suggested that growth inhibition with dose dependence among all treatment groups (Figure 3d). CaIM induced the most pronounced reduction in cell viability, far exceeding the effects of BP or CaO_2_ alone. BP displayed the lowest cytotoxicity, which can be attributed to excellent biocompatibility and the nontoxic nature of phosphate degradation products. The slight concentration‐dependent viability decrease observed for BP may result from osmotic perturbations across the cellular membrane. By integrating Ca^2^^+^ overload and ROS generation, CaIM achieved a synergistic enhancement of cytotoxicity that could not be attained by either component alone. Flow cytometric analysis further confirmed that CaIM effectively induced apoptosis, with both early and late apoptotic fractions markedly increased compared with other treatment groups (Figure S7). Meanwhile, wound healing assays revealed that CaIM significantly suppressed cell migration, indicating the potential to interfere with the invasive and metastatic tumor cells (Figure S8). These findings collectively validated the synergistic antitumor effects of CaIM in inducing cell death, apoptosis, and inhibiting migration.

Mitochondrial membrane potential was a crucial indicator of mitochondrial integrity and energy metabolism. To verify mitochondrial impairment, JC‐1 staining was performed. Cells treated with CaIM displayed a pronounced shift from red to green fluorescence, indicating the substantial loss of mitochondrial membrane potential (Figure S9). Numerous studies have demonstrated that mitochondria are highly sensitive to oxidative stress, which serves as a key regulator of intracellular calcium homeostasis [22]. ROS bursts are a central trigger for mitochondrial damage and apoptosis [23]. Loss of mitochondrial membrane potential was not only a hallmark of mitochondrial damage but also a critical trigger for apoptosis, leading to cell death. Bio‐TEM imaging further revealed swollen mitochondria and disrupted cristae, hallmark features of severe mitochondrial structural damage (Figure 3e). The mitochondrial dysfunction induced by CaIM treatment was a critical event triggered by calcium overload and oxidative stress. This created a self‐amplifying feedback loop, where mitochondrial dysfunction further potentiated calcium dysregulation and oxidative stress, ultimately triggering apoptotic signaling pathways. Therefore, the potent antitumor effects of CaIM were largely attributed to the synergistic interplay between calcium overload and oxidative stress, which converged to induce mitochondrial dysfunction and apoptosis.

### 2.3 Robust Cellular Calcification

In clinical practice, tumor calcification necrosis has frequently been observed. Tumor calcification following radiotherapy and chemotherapy is considered a favorable prognostic factor in hepatocellular carcinoma (HCC). However, the formation of tumor calcification in clinical settings is characterized by a protracted timeline and an exceedingly low incidence rate. It is widely acknowledged that calcification cannot occur within physiological ranges of ion concentrations. Instead, it is contingent upon the presence of elevated concentrations of mineral ions such as Ca^2^^+^ (>10 mm) and phosphate ions within the tumor microenvironment. These observations prompt us to investigate whether the exogenous introduction of calcium and phosphate ions mediated by the CaIM can effectively induce tumor cell calcification. Bio‐TEM imaging revealed that cells with CaIM treatment developed extensive ultrastructural alterations, including the formation of mineralized vesicle‐like structures, a roughened cell surface, and progressive cellular shrinkage, all of which were characteristic features associated with cell calcification (Figure 3f,g). Notably, these mineralized vesicles resemble matrix vesicles reported in pathological biomineralization processes, suggesting an active intracellular mineral nucleation mechanism. Elemental mapping further confirmed a substantial intracellular enrichment of Ca signals exclusively in CaIM‐treated cells, whereas negligible calcium accumulation was detected in the control or BP groups (Figure 3g). This observation indicated that CaIM effectively disrupts and drives abnormal mineral accumulation. Alizarin Red S staining further visualized extensive calcium salt deposition in the CaIM group (Figure 3h). The above results elucidate the calcification patterns of cells following CaIM treatment across multiple scales, encompassing the formation of nanoscale mineralized vesicles, alterations in micron‐scale cellular structures/elemental distributions, and the emergence of macroscopic mineral nodules. These distinct and progressively intensifying phenotypic manifestations represent significant consequences of tumor cell calcification induced by sustained calcium overload, rather than being passive byproducts of cell death.

### 2.4 CaIM‐Induced Immune Modulation

Macrophages are a class of multifunctional immune cells capable of undergoing morphological and functional differentiation in response to changes in the microenvironment. This polarization process is of critical importance for activating anti‐tumor immunity. Previous studies have confirmed that the polarization of macrophages is regulated by the cellular uptake of extracellular Ca^2^^+^ [24]. Elevated Ca^2^^+^ activates p38/MAPK and NF‐κB pathways to promote M1 polarization, whereas Ca^2^^+^ reduction favors M2 phenotypes [25]. Macrophage polarization analysis demonstrated that CaIM markedly increased the proportion of CD86 (M1 macrophage maker) while reducing CD206 (M2 macrophage maker) populations (Figure 3i). This shift indicated that CaIM‐mediated calcium overload actively remodeled the tumor immune microenvironment rather than merely killing tumor cells in isolation. Collectively, CaIM simultaneously induced intracellular calcium overload, triggered biomineralization, and reprogrammed immune responses, thereby exerting dual antitumor functions. Tumor cells were directly eliminated through Ca^2^^+^ overload‐driven calcification and mitochondrial collapse, while immune suppression within the tumor microenvironment was reversed via calcium signal‐dependent macrophage polarization. These findings established CaIM as a calcium‐based therapeutic platform that integrated calcification therapy with immune modulation, offering a mechanistically distinct and synergistic antitumor strategy.

### 2.5 Evaluation of Therapeutic Efficacy In Vivo

**FIGURE 4:**
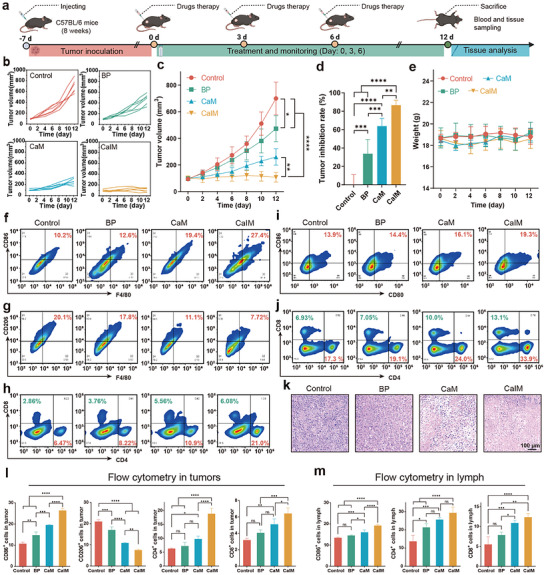
Therapeutic evaluation and immune profiling in vivo. (a) Schematic of the treatment schedule presenting tumor inoculation, drug administration, monitoring time points, and tissue collection. (b) Tumor growth curves of individual mice in each treatment group. (c) Average tumor volume during the treatment period. (d) Tumor inhibition rates at the study endpoint. (e) Body weight monitoring during the treatment period. (f, g) Flow cytometry plots showing CD86^+^ macrophages (f) and CD206^+^ macrophages (g) in tumors for different treatment groups. (h) Flow cytometry plots of CD4^+^ T cells and CD8^+^ T cells in tumors for different treatment groups. (i) Flow cytometry plots showing CD86^+^ macrophages in lymph nodes for different treatment groups. (j) Flow cytometry plots of CD4^+^ T cells and CD8^+^ T cells in lymph nodes for different treatment groups. (k) Representative H&E staining images of tumor sections from each treatment group. (l) Quantification of macrophage populations and T cell populations in tumors. (m) Quantification of macrophage populations and T cell populations in lymph nodes. ^*^ *p* < 0.05; ^**^ *p* < 0.01; ^***^ *p* < 0.001; ^****^ *p* < 0.0001; n.s., not significant, *n* = 6.

Based on the potent antitumor activity observed in vitro, a subcutaneous tumor‐bearing mouse model was established to systematically evaluate the therapeutic efficacy of CaIM in vivo. Mice were randomly assigned to four groups, including Control, BP, CaM, and CaIM. Intratumoral injections were administered following the predetermined schedule and dynamic monitoring throughout the treatment period (Figure 4a). Tumor growth curves indicated that BP exerted only minimal tumor suppressive effects, whereas CaO_2_ achieved moderately improved inhibition due to elevated intracellular Ca^2^^+^. In contrast, CaIM treatment produced the most pronounced tumor growth inhibition, with tumor volumes remaining consistently the lowest among all groups during the entire treatment course, indicating a superior in vivo therapeutic outcome (Figure 4b,c). At the treatment endpoint, tumors were excised and measured. Based on the final tumor weight, the tumor inhibition rate of CaIM exceeded 80%, which was significantly higher than that of the BP and CaM groups (Figure 4d).

To evaluate biosafety, hemocompatibility, and systemic toxicity were carefully assessed. No visible hemolysis was observed even at CaIM concentrations as high as 800 µg/mL, demonstrating excellent blood compatibility (Figure S10). Body weights of mice in all treatment groups remained stable throughout the experimental period, with no statistically significant differences observed (Figure 4e). Histological examination of major organs, including heart, liver, spleen, lung, and kidney, revealed no detectable pathological abnormalities following CaIM treatment (Figure S11). Furthermore, serum biochemical indices and hematological parameters remained within normal physiological ranges after CaIM administration, collectively confirming favorable systemic biosafety and biocompatibility (Figures S12 and S13). Collectively, these results demonstrated that CaIM achieved robust antitumor efficacy in vivo while maintaining favorable biocompatibility and safety.

### 2.6 Immune Activation and Tumor Microenvironment Remodeling

Building on the in vitro findings that CaIM induced calcium overload and oxidative stress effectively reprogrammed macrophages toward a pro‐inflammatory phenotype, we next explored whether CaIM could similarly remodel the tumor immune microenvironment in vivo. As shown in Figure 4f,g, CaIM markedly increased the proportion of M1‐like macrophages (CD86^+^F4/80^+^) while decreasing M2‐like macrophages (CD206^+^F4/80^+^) in tumor tissues, indicating reversal of the immunosuppressive microenvironment. This immune reprogramming was closely linked to the central role of Ca^2^^+^ signaling in macrophage phenotype regulation, as elevated intracellular Ca^2^^+^ has been reported to activate p38/MAPK and NF‐κB pathways, thereby promoting M1 polarization [25]. The shift toward an M1‐dominant macrophage landscape created a more immunostimulatory tumor microenvironment, which in turn facilitated enhanced antigen presentation and T cell activation. Consistently, CaIM significantly promoted dendritic cell (DC) maturation in secondary lymphoid organs, indicating improved antigen uptake and processing (Figure S14). Simultaneously, both CD4^+^ and CD8^+^ T cell infiltration increased substantially following CaIM treatment (Figure 4h–j and Figure S15). Quantitative analysis further confirmed statistically significant elevations in these effector immune populations (Figure 4l,m). Such elevated T‐cell infiltration was typically associated with the transition of tumors from an “immune‐cold” to an “immune‐hot” state, reflecting a shift toward an immune‐responsive microenvironment that favored effective antitumor immunity. Immune phenotype transformation was widely associated with improved antitumor immune surveillance and therapeutic responsiveness. Histological assessments supported these immunological findings, revealing pronounced nuclear shrinkage and structural loosening in CaIM‐treated tumors, consistent with enhanced immune‐mediated tumor destruction (Figure 4k). In addition, immunofluorescent staining of TUNEL, Ki67, and immune‐related markers (such as CD80, CD86, CD206, CD4, and CD8) further confirmed enhanced apoptosis, suppressed proliferation, and strong immune activation (Figure S16–S20). Calcium dysregulation and oxidative stress not only directly damaged tumor cells but also reshaped the tumor immune microenvironment. CaIM effectively reprogrammed the microenvironment, converting an immunosuppressive tumor milieu into an activated state, thereby amplifying antitumor immunity and supporting durable therapeutic efficacy.

### 2.7 Stable Tumor Biomineralization With Significant Diagnostic and Therapeutic Implications

**FIGURE 5:**
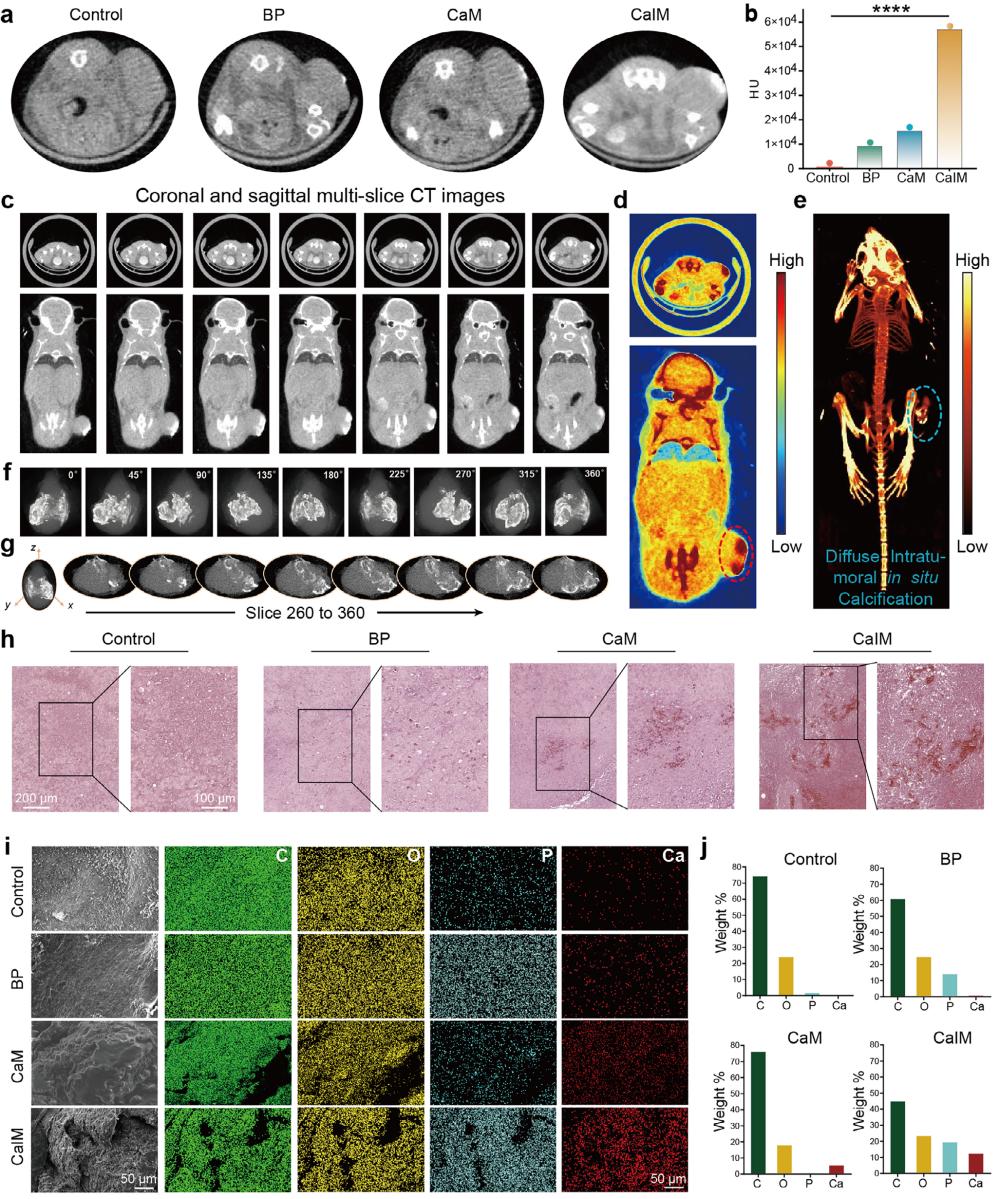
Visualization and characterization of CaIM‐induced tumor calcification. (a) Representative CT images of tumor sites collected 7 days after treatment with the indicated formulations. (b) Quantified signal intensities extracted from CT images for each group. (c) Multiplanar CT images of tumors acquired in sagittal and coronal planes, visualizing the spatial distribution of CaIM‐induced calcification. (d) Whole‐body 3D CT rendering of treated mice, visualizing the global distribution and anatomical location of calcification. (e) 3D CT volume reconstruction of treated mice, highlighting localized tumor calcification. (f) Rotational CT imaging of tumors acquired from 0° to 360° at 45° intervals. (g) Serial CT layer scanning of tumor tissues across consecutive slices. Tumor tissues corresponding to the rotational views. (h) Alizarin Red staining of tumor sections from each treatment group to visualize calcium nodes. (i) SEM images and EDS elemental mapping of C, O, P, and Ca in tumor tissues following treatment. (j) Elemental composition (wt.%) of tumor tissues calculated from EDS analyses. ^****^ *p* < 0.0001, *n* = 3.

Tumor calcification has been recognized as a favorable indicator, correlating with improved therapeutic outcomes [3, 26]. Notably, CaIM supplies both Ca^2^^+^ through CaO_2_ decomposition and phosphate species through BP degradation, providing the essential components required for calcification formation in vivo. Considering the pronounced intracellular calcification observed in vitro and the strong therapeutic efficacy demonstrated in vivo, we further carried out in‐depth validation to confirm the tumor calcification in vivo. Small‐animal CT imaging was conducted to noninvasively visualize mineral deposition. CaIM injection produced strong high‐density signals within the tumor region, indicating significant calcium salt deposition (Figure 5a). Compared with the control, BP, and CaM groups, the CaIM group exhibited markedly elevated CT radiodensity, reflecting a higher degree of mineralization (Figure 5b). CT imaging with sagittal and coronal planes was performed to monitor and observe the biomineralization, revealing progressive radiodensity induced by CaIM (Figure 5c). Three‐dimensional CT reconstruction further visualized extensive calcium deposition within the tumor (Figure 5d,e). Meanwhile, the 0°–360° rotational imaging confirmed the continuity of the calcified regions (Figure 5f). The corresponding video of the 0°–360° rotational CT imaging was presented in Video S1. Continuous CT layer scanning was additionally employed to assess the distribution of calcium salts across tumor slices. High‐density regions were consistently detected throughout slices 260 to 360 in the CaIM group, suggesting widespread rather than focal mineralization (Figure 5g). Alizarin Red staining further corroborated these results and revealed substantial calcium salt deposition throughout the tumor, with an expanded calcification area in the CaIM group (Figure 5h). SEM‐EDS was used to perform elemental analysis to clarify elemental composition and confirm distribution characteristics of deposited minerals (Figure 5i). A pronounced calcium signal was observed on the tumor surface following CaIM treatment, with calcium content reaching 9 wt.%, exceeding that of other groups. Concurrent enrichment of phosphorus was also detected, indicating that the released Ca^2^^+^ combines with phosphate species generated from BP degradation to form calcium minerals. Observed tumor mineralization corresponded to the final product facilitated by the decomposition of BP and Ca^2^^+^ released from calcium peroxide, along with the stressed tumor microenvironment. (Figure 5i,j). BP nanosheets undergo progressive biodegradation through surface oxidation and hydrolysis in the presence of water, dissolved oxygen, and reactive oxygen species, generating biocompatible phosphate species (H_2_PO_4_^−^/HPO_4_^2^^−^/PO_4_^3^^−^) as the terminal degradation products [27, 28]. Critically, this degradation process is substantially accelerated under the acidic, H_2_O_2_‐enriched tumor microenvironment actively generated by CaO_2_ decomposition, establishing a self‐reinforcing biomineralization cascade: CaO_2_ decomposition lowers local pH and elevates H_2_O_2_, which in turn accelerates BP oxidative degradation and phosphate release; the released PO_4_^3^^−^ then combines with concurrently liberated Ca^2^^+^ to drive hydroxyapatite nucleation in situ. Quantitative EDS analysis (Figure 5j) revealed that the P content in CaIM‐treated tumour tissues reached 19.46 wt.%, substantially exceeding that of the BP‐alone group, and concurrent Ca enrichment was detected exclusively in the CaIM group, confirming that BP‐degradation‐derived phosphate actively participates in intratumoral biomineralization in vivo rather than being a passive degradation byproduct. From a clinical transformation perspective, this robust intratumoral calcification not only reflected a strong therapeutic response but also offered a highly visible imaging signature that can aid in distinguishing treatment‐responsive lesions during clinical follow‐up.

### 2.8 Proteomic Profiling Reveals the Synergistic Regulatory Network

**FIGURE 6:**
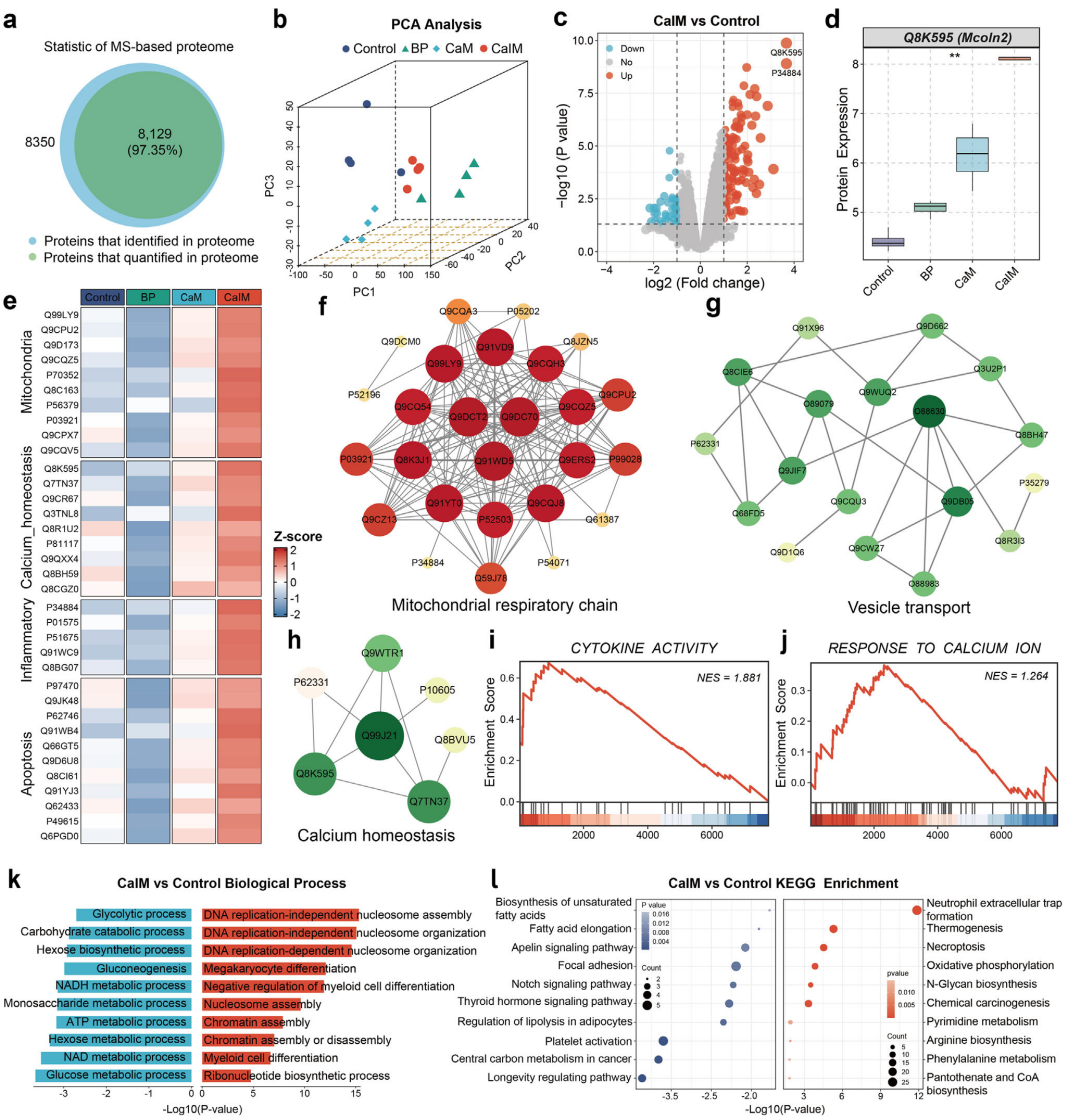
Proteomic profiling revealed that CaIM induced coordinated calcium dysregulation, mitochondrial stress, and immune‐related pathway activation in tumor tissues. (a) Overview of the proteomic dataset containing 8543 proteins identified and 8311 reliably quantified proteins. (b) Principal component analysis (PCA) demonstrating clear separation among groups, indicating distinct proteomic remodeling. (c) Volcano plot suggesting extensive differential protein expression in the CaIM group relative to control. (d) Box plot exhibiting expression of MCOLN2, a lysosomal Ca^2^^+^ channel, markedly elevated in the CaIM group. (e) Heatmap of representative proteins associated with mitochondrial function, calcium homeostasis, inflammation, and apoptosis, illustrating broad proteomic shifts. (f–h) Protein‐protein interaction (PPI) networks highlighting three major modules altered by CaIM, including mitochondrial respiratory chain (f), vesicle transport (g), and calcium homeostasis (h). (i, j) GSEA enrichment displayed the activation of cytokine‐related pathways (i) and calcium‐response pathways (j) in CaIM groups. (k) GO enrichment revealing upregulation of immune‐related and stress‐responsive biological processes and downregulation of glucose and ATP metabolic pathways. (l) KEGG analysis identifying suppression of tumor progression pathways and enrichment of mitochondrial stress, necroptosis, oxidative phosphorylation, and other immune‐associated pathways. ^**^ *p* < 0.01, *n* = 4.

To elucidate the molecular basis underlying antitumor and immunomodulatory activities of CaIM, TMT‐based quantitative proteomics was performed on tumor tissues collected from the control, BP, CaM, and CaIM groups. A total of 8543 proteins were identified, among which 8311 were reliably quantified (Figure 6a). Principal component analysis (PCA) revealed clear separation among the four treatment groups, indicating that each intervention induced a distinct proteomic signature within the TME (Figure 6b). Differential expression analysis identified massive molecular alterations across treatment groups (Figure 6c and Figure S21). Compared with the control group, CaO_2_ induced 180 differentially expressed proteins, whereas CaIM triggered 602, demonstrating the markedly enhanced molecular remodeling capacity of the composite system (Figure S22). Consistently, heatmap visualization further illustrated the extensive proteomic shifts induced by CaIM (Figure S22). Among the significantly upregulated proteins, two stood out as mechanistically relevant were identified in CaIM group, MCOLN2 (Q8K595) and CBR1 (P34884) (Figure 6d and Figure S23). MCOLN2, a Ca^2^^+^ channel, is critically involved in lysosomal Ca^2^^+^ efflux, endolysosomal trafficking, and macrophage M1 polarization [29]. MCOLN2 with high expression supports the notion that CaIM‐induced Ca^2^^+^ dynamics actively participate in immune activation. CBR1, an NADPH‐dependent oxidoreductase, regulates cellular redox balance and drug‐responsive apoptotic signaling [30]. Upregulated CBR1 indicated heightened oxidative stress consistent with ROS generation by CaIM.

Notably, proteins associated with inflammatory signaling, apoptosis execution, calcium homeostasis, and mitochondrial electron transport were markedly elevated in the CaIM group (Figure 6e). These shifts align with our mechanistic observations that CaIM induces Ca^2^^+^ overload, mitochondrial injury, and immune activation, suggesting the presence of a coordinated response network within tumors. Using the STRING database, protein–protein interaction (PPI) analysis further uncovered three mechanistically interconnected modules, including mitochondrial respiratory chain, vesicle trafficking, and calcium homeostasis (Figure 6f,h). Upregulation of respiratory chain components (Complex I‐IV) indicates mitochondrial hyperactivation under Ca^2^^+^ and ROS stress, a hallmark associated with immunogenic cell death [31]. Enhanced vesicle transport reflects the intracellular trafficking pattern of CaIM, which was from endocytosis through lysosomes to mitochondria, consistent with our in vitro imaging evidence. The broad elevation of Ca^2^^+^ regulating proteins (e.g., MCOLN2, S100 family) confirms extensive remodeling of intracellular Ca^2^^+^ fluxes. CaIM performed biological effects through a coordinated network in which Ca^2^^+^ influx caused disruption of calcium homeostasis, triggering mitochondrial stress, ultimately amplifying inflammatory signaling and activating immune pathways.

In addition, GSEA analysis reinforced this mechanism. CaIM exhibited significant enrichment in cytokine activity (NES = 1.881) and response to calcium ions (NES = 1.264), suggesting synchronous enhancement of Ca^2^^+^ responsive pathways and cytokine‐mediated immune activation (Figure 6i,j). GO enrichment revealed that CaIM upregulated pathways linked to myeloid differentiation, immune activation, and programmed cell death, while downregulating glucose metabolism and ATP metabolism (Figure 6k and Figures S24 and S25). In contrast, CaO_2_ alone induced weaker metabolic and immune perturbations. KEGG enrichment further substantiated the superior regulatory capacity of CaIM (Figure 6l and Figure S26). Tumor progression pathways were significantly downregulated, including focal adhesion, Notch signaling, and platelet activation, whereas immune‐related pathways were markedly enriched, such as oxidative phosphorylation, necroptosis, mitochondrial stress, and neutrophil extracellular trap formation (Figure 6l and Figure S24). Although CaO_2_ also upregulated certain stress‐related pathways, only CaIM achieved the combinatorial activation of Ca^2^^+^ disturbance, mitochondrial dysfunction, and immune reprogramming, reflecting unique multi‐mechanistic therapeutic advantages.

Taken together, proteomic analysis revealed that CaIM displayed therapeutic effects through a complicated regulatory network in which Ca^2^^+^ homeostasis disruption induced mitochondrial stress, triggered metabolic collapse, and initiated immune activation, culminating in synergistic antitumor responses. Integrated mechanisms explained the strong therapeutic efficacy observed in vitro and in vivo, providing compelling molecular evidence that CaIM drives a synergistic regulatory network to exert potent antitumor effects.

### 2.9 MCOLN2 Emerges as a Calcium‐Responsive Molecular Mediator Linking Ca^2^^+^ Perturbation to Immunomodulatory Networks

**FIGURE 7:**
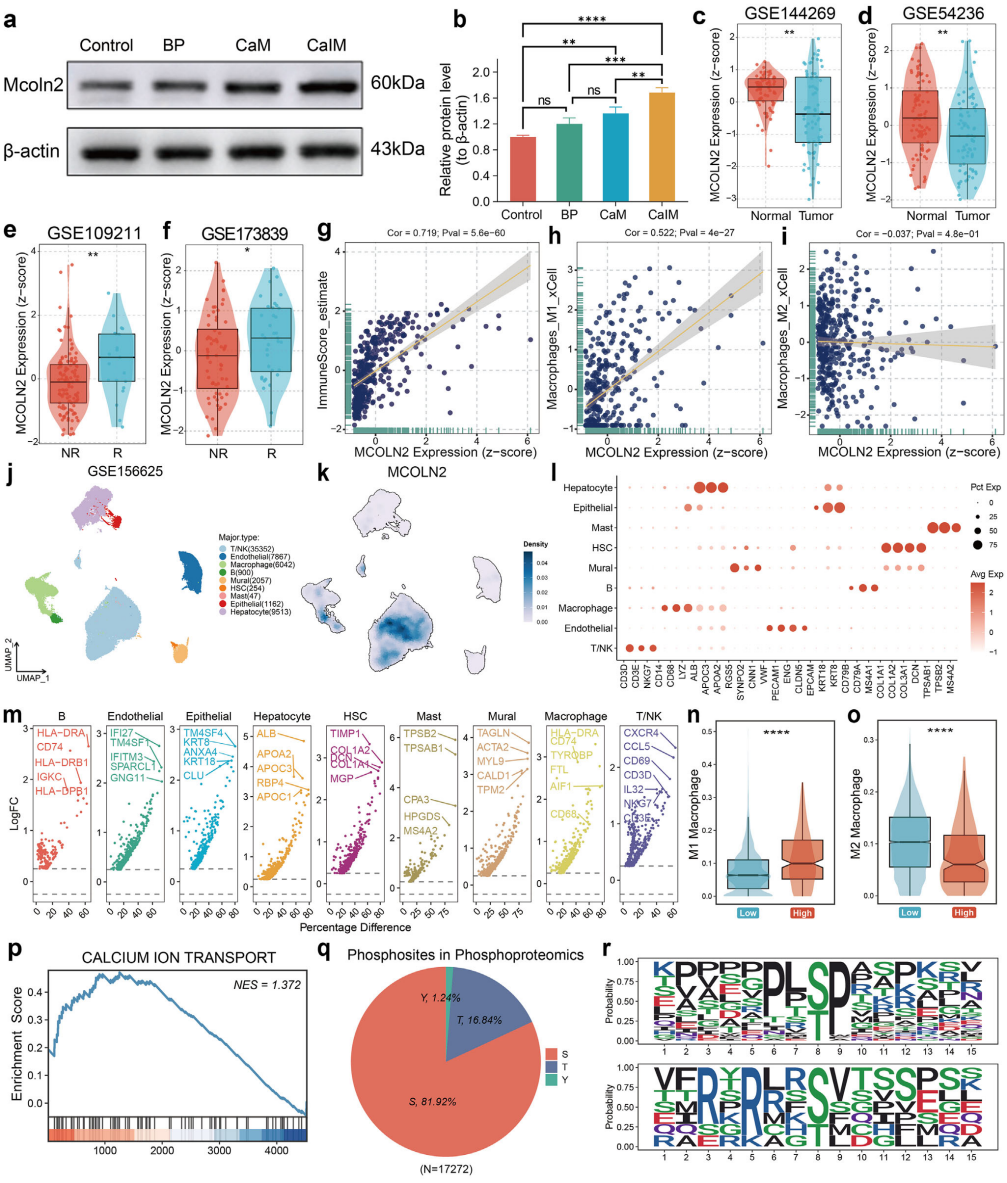
MCOLN2 was identified as a calcium‐responsive regulatory hub linking Ca^2^^+^ overload to immune remodeling in tumors. (a) Western blot analysis displaying upregulation of the Ca^2^^+^‐responsive lysosomal channel MCOLN2 across treatment groups, with the highest induction observed after CaIM administration. (b) Quantification of MCOLN2 protein levels normalized to β‐actin, *n* = 3. (c, d) MCOLN2 expression patterns in normal and tumor tissues from public liver cancer datasets, such as GSE144269, *n* = 140 (c) and GSE54236, *n* = 161 (d), indicating protective factors with potential clinical value. (e, f) MCOLN2 expression in responders and non‐responders from immunotherapy rdatasets, such as GSE109211, *n* = 140 (e) and GSE173839, *n* = 105 (f), where higher expression was associated with immunotherapy responsiveness. (g–i) Correlation analyses proved that MCOLN2 expression aligned strongly with immune activation, including positive association with ImmuneScore (g) and M1 macrophage infiltration (h), while minimal association with M2 macrophage infiltration (i). The xCell algorithm was employed to calculate ImmuneScore, M1 macrophage infiltration, and M2 macrophage infiltration. (j) The single‐cell RNA‐seq data were used for dimensionality reduction and clustering. UMAP visualization of identifying the major cell populations. (k) The distribution of MCOLN2 expression among distinct cell populations. MCOLN2 expression enriched in immune‐related cell types, particularly macrophages and T/NK cells. (l, m) Classic cell markers (l) and differential expression profiles (m) defining major stromal and immune cell subsets. (n, o) Distribution of M1 macrophages (n) and M2 macrophages (o) infiltration stratified by MCOLN2 expression level, suggesting macrophage polarization patterns associated with MCOLN2. (p) In single cell level, GSEA enrichment analysis highlighting calcium transport pathways in cells with high MCOLN2 expression, consistent with calcium‐responsive signaling. (q) Composition of identified phosphosites from phosphoproteomic profiling, revealing extensive phosphorylation regulation across Ser, Thr, and Tyr residues. (r) Enriched phosphorylation motifs in CaIM upregulated phosphopeptides, representing kinase activation signatures associated with calcium signaling and immune signaling. ^*^ *p* < 0.05; ^**^ *p* < 0.01; ^***^ *p* < 0.001; ^****^ *p* < 0.0001; n.s., not significant.

Excessive Ca^2^^+^ influx and H_2_O_2_ generated by CaIM initiate a cascade of calcium channel dysregulation and intracellular calcium overload, ultimately leading to mitochondrial injury, tumor calcification, and cell death. To identify the molecular node connecting calcium dyshomeostasis and immune activation, mult‐omics analysis was conducted. MCOLN2 served as a TRPML2 calcium channel protein, which emerged as one of the most significantly upregulated proteins in the TMT proteomic profiling. Western blotting confirmed a pronounced elevation of MCOLN2, especially in CaIM‐treated tumors, highlighting MCOLN2 as a primary Ca^2^^+^ responsive molecule that amplified calcium‐dependent stress signaling (Figure 7a,b). Based on GSE144269 and GSE54236 cohorts, expression profiling revealed that MCOLN2 was highly expressed in normal liver tissue but markedly suppressed in tumors, suggesting that MCOLN2 is a protective protein during tumor progression (Figure 7c,d). In immunotherapy cohorts (GSE109211 and GSE173839), treatment responders displayed significantly higher MCOLN2 expression, linking MCOLN2 to improved immunotherapy responsiveness (Figure 7e,f). Consistently, MCOLN2 expression displayed strong positive correlations with ImmuneScore (R = 0.719) and M1 macrophage infiltration (R = 0.522), but minimal association with M2 macrophages (R = −0.037), indicating that MCOLN2 promoted immune activation (Figure 7g,i).

To further delineate the cellular origin and immunological relevance of MCOLN2, single‐cell RNA sequencing data were processed and employed for high‐resolution clustering and cell annotation (Figure 7j). MCOLN2 exhibited enriched expression in immune cells, such as T/NK cells and macrophages (Figure 7k). DotPlot and volcano plot further confirmed cell subpopulation and differential expression genes among distinct cell types (Figure 7l,m). At the single‐cell level, elevated MCOLN2 expression exhibited markedly increased infiltration of M1 macrophages and reduced M2 macrophages, indicating MCOLN2 promoted macrophage reprogramming (Figure 7n,o). GSEA demonstrated significant enrichment of calcium ion transport (NES = 1.372) in cells with high MCOLN2 expression (Figure 7p). Therefore, Ca^2^^+^ overload was an upstream trigger for MCOLN2 elevation and the ensuing immune reprogramming. To further decipher the upstream kinase signaling landscape under Ca^2^^+^ overload, global phosphoproteomic profiling was conducted across treatment groups. A total of 17,272 phosphosites were identified, comprising Ser (81.92%), Thr (16.84%), and Tyr (1.24%), a distribution characteristic of kinase regulatory networks (Figure 7q). Notably, phosphorylation changes were more extensive and sensitive than total protein abundance changes, suggesting that CaIM triggered rapid signaling amplification via phosphor regulatory networks (Figures S27 and S28). Motif‐X analysis revealed two prominently enriched phosphorylation motifs in CaIM‐upregulated phosphopeptides (Figure 7r). The SP/SxP motif corresponds to substrates of proline‐directed kinases, including MAPKs and CDKs, suggesting activation of stress signaling, cell cycle perturbation, and apoptosis under calcium overload and oxidative stress [32, 33]. The SxxS/SxS motif, typically targeted by CK2, CaMK, and IKK kinases, reflects potent Ca^2^^+^ dependent and inflammation‐related phosphorylation, consistent with NF‐κB activation, inflammatory amplification, and immune remodeling [34, 35]. Collectively, these phosphoproteomic signatures validated MCOLN2 as a central effector embedded within a coordinated kinase activation program that links calcium dynamics to macrophage polarization, immune activation, and the potent antitumor effects of CaIM.

## 3 Conclusions

Calcium signaling has long been recognized as a central regulator of cell fate, metabolism, and immunity [7, 36, 37]. Due to the lack of efficient tools driving Ca^2^^+^ overload and structural biomineralization, exploiting tumor calcification for therapeutic purposes remains challenging. Conventional CaO_2_‐based platforms release intermittent bursts of Ca^2^^+^ and H_2_O_2_, generating oxidative stress and perturbing ionic balance within tumors [38, 39]. The intrinsic instability of CaO_2_ decomposition leads to poorly controlled Ca^2^^+^ release, preventing any precise coordination with intracellular stress pathways [40]. Moreover, the absence of an endogenous phosphate source prevents the formation of stable calcium deposition, rendering sustained intratumoral calcification difficult to attain [41]. Meanwhile, oxidative stress is required to involve and accelerate the biosynthesis of calcium minerals [42, 43]. Based on these challenges and mechanisms of calcium mineral biosynthesis, we customized a calcium nanomodulator, CaIM, which was synthesized via in situ growth of CaO_2_ on BP nanosheets, rather than simple physical loading, yielding crystalline CaO_2_ nanoparticles uniformly anchored on the BP matrix. In situ growth‐derived CaIM provided sustained Ca^2^^+^ supply, endogenous phosphate generation, and oxidative stress amplification. Tumor calcification has traditionally been regarded as a passive physicochemical consequence of tissue necrosis, which is stochastic, poorly controllable, and strongly dependent on the severity of tissue injury [44]. Our findings proposed a fundamentally distinct therapeutic paradigm as an actively orchestrated outcome of calcium signaling. Steady Ca^2^^+^ and H_2_O_2_ release from CaO_2_ enabled synchronized calcium overload and oxidative stress within tumors. BP‐derived PO_4_^3^^−^ allowed stable calcium‐phosphate biomineralization in vivo. Localized ionic supersaturation was favorable for mineral nucleation, enabling the formation of widespread, homogeneous, and structurally stable calcium phosphate deposits, rather than focal or incidental mineral remnants. Tumor calcification should be viewed not only as a terminal marker of tissue necrosis, but also as an engineerable point within a therapeutic strategy. By strategically redirecting intracellular Ca^2^^+^ flux and enhancing inter‐organelle communication, it is possible to simultaneously enhance both tumor calcification and cell death. This conceptual advancement has broader implications and addresses a key gap in tumor calcification. Further, extending Ca^2^^+^ flux therapeutic paradigm to other solid tumors may broaden its clinical applicability. Collectively, CaIM offers a conceptually distinct advance over previously reported calcium‐based and calcification‐inducing platforms by uniting in situ‐grown crystalline CaO_2_ anchoring, synchronized endogenous phosphate supply, and redox amplification within a single programmable nanoplatform, thereby overcoming both the burst‐release instability of physically assembled CaO_2_ formulations and the phosphate‐deficiency bottleneck that has precluded durable intratumoral hydroxyapatite biomineralization.

A distinguishing feature of CaIM was driving extensive intratumoral calcification, far exceeding what has been observed with CaO_2_ or BP alone. Compared with previously reported calcium‐based strategies, CaIM also exhibited a markedly more intense and widespread tumor calcification phenotype. CaIM induced mineralization that markedly enhanced CT contrast, providing a clear and quantifiable imaging signature for treatment response assessment. From a clinical perspective, tumor calcification has been repeatedly associated with improved clinical prognosis [45]. Here, calcification not only served as a reliable indicator of therapeutic response but also enhanced the radiographic visibility of the tumor region. CaIM integrated therapeutic efficacy, imaging enhancement, and prognostic value into a unified platform, underscoring the potential for next‐generation cancer therapy. Beyond structural calcification, CaIM induced a coordinated biological cascade in which calcium overload and oxidative stress converged on mitochondrial dysfunction and immune activation. Calcium overload disrupted mitochondrial calcium homeostasis, amplified reactive oxygen species, and triggered mitochondrial collapse, providing a mechanistic basis for enhanced tumor cell apoptosis compared with CaO_2_ or BP alone. Importantly, calcium overload also functioned as an immunomodulatory signal, promoting dendritic cell maturation, macrophage polarization toward the M1 phenotype, and increasing infiltration of cytotoxic T lymphocytes, thereby reshaping the tumor microenvironment toward an immune‐responsive state. Therefore, CaIM, promoting calcium signaling, displayed a critical molecular bridge in governing tumor cell fate.

Integrated multi‐omics analyses identified MCOLN2 as a Ca^2^^+^ release channel, functioning as a central molecular mediator linking Ca^2^^+^ perturbation with immune remodeling. MCOLN2 is known to regulate lysosomal Ca^2^^+^ efflux, organelle crosstalk, and inflammatory signaling [46, 47]. Proteomics and western blot suggested that MCOLN2 was rapidly and robustly upregulated upon CaIM treatment, occurring before detectable calcium‐phosphate deposition. This temporal precedence positioned MCOLN2 as an early predictive biomarker for tumor calcification, providing a molecular tool for assessing calcification sensitivity and monitoring therapeutic response. Beyond materials engineering, this work reframes tumor calcification as an actively orchestrated immune‐remodeling process rather than a passive cytotoxic endpoint and establishes multi‐omics‐guided identification of MCOLN2 as a calcium‐responsive molecular mediator that couples biomineralization kinetics with macrophage polarization and immune activation for integrated treatment monitoring and outcome prediction. Future studies will evaluate CaIM in clinically relevant delivery systems and pursue systematic functional validation of MCOLN2 to advance its translational potential for calcification‐guided therapy monitoring.

Overall, CaIM was employed to establish a therapeutic paradigm based on tumor calcification that integrates calcium stress, oxidative stress, mitochondrial dysfunction, as well as calcium‐triggered immune activation. The identification of MCOLN2 introduced a new biomarker with strong potential for evaluating tumor calcification and clinical outcome.

## 4 Experimental Section

### 4.1 Ethics Approval and Consent to Participate

The animal experimental protocol was approved by the Animal Ethics Committee of Xi'an Jiaotong University (Approval Number: XJTUAE20‐2419). A full description of the materials and methods, as well as all experimental details are provided in the Supporting Information.

### 4.2 Statistical Analysis

All experimental data obtained from at least three independent replicates are expressed as mean ± standard deviation (SD). The details of replicates and error bar definitions are provided in the figure legends. For statistical comparisons, two‐tailed Student's *t*‐tests were applied between two groups, whereas multiple groups were evaluated using one‐way ANOVA with Tukey's test. A *p*‐value less than 0.05 was regarded as statistically significant. All statistical evaluations were conducted with GraphPad Prism and R program.

## Author Contributions

**Long Liu** contributed to conceptualization, methodology, and Writing – review and editing. **Shuang Bai** and Long Liu contributed to data curation and investigation. Long Liu, **Peng Li**, and **Zhixiang Lu** contributed to conceptualization, methodology, visualization, and writing – original draft. **Shuqin Xu**, **Juanjuan Wang**, **Kai Ma**, **Haodong Yu**, **Can Zhou**, and **Hu Chen** contributed to writing – review and editing. Shuang Bai, **Yi Lyu**, and **Gang Liu** contributed to conceptualization, funding, writing – review and editing. All authors have read the final manuscript and approved it for publication.

## Conflicts of Interest

The authors declare no conflicts of interest.

## Supporting information




**Supporting File 1**: advs76642‐sup‐0001‐SuppMat.docx.

**Supporting File 2**: advs76642‐sup‐0002‐VideoS1.mp4.

## Data Availability

The data that support the findings of this study are available in the supplementary material of this article.
