# Supplementary material for: A Programmable Calcification Nanoplatform for Loco‐Regional Calcification‐Immune Hepatocellular Carcinoma Therapy
Source: Adv Sci (Weinh). 2026 Jul 20:e76642. Online ahead of print. doi: 10.1002/advs.76642 (PMC13383153; doi:10.1002/advs.76642)
Supplement: Supplementary file 1 — Supporting File 1: advs76642‐sup‐0001‐SuppMat.docx. [file ADVS-9999-e76642-s002.docx]

**Supporting Information**

**A programmable calcification nanoplatform for Loco-regional Calcification-Immune Hepatocellular Carcinoma Therapy**

*Long Liu ^a, b, c^* **^#^***, Peng Li ^d^* **^#^***, Zhixiang Lu ^e^* **^#^***, Shuqin Xu ^a, b, c^, Juanjuan Wang ^a, b^, Kai Ma ^a, b^, Haodong Yu ^a, b^,* *Can Zhou ^f^, Hu Chen ^a, b^, Gang Liu ^e*^, Yi Lyu ^a, b, c*^, Shuang Bai ^a, b*^*

^a^ Shaanxi Province Center for Regenerative Medicine and Surgery Engineering Research, the First Affiliated Hospital of Xi’an Jiaotong University, Xi’an 710061, China.

^b^ National Local Joint Engineering Research Center for Precision Surgery & Regenerative Medicine, the First Affiliated Hospital of Xi’an Jiaotong University, Xi’an 710061, China

^c^ Department of Hepatobiliary Surgery, the First Affiliated Hospital of Xi’an Jiaotong University, Xi’an 710061, China.

^d^ Institute of Regenerative and Reconstructive Medicine, Med-X Institute, The First Affiliated Hospital of Xi’an Jiaotong University, Xi’an 710061, China.

^e^ State Key Laboratory of Cellular Stress Biology, Innovation Center for Cell Biology, School of Life Sciences, Xiamen University, Xiamen 361102, China.

^f^ Department of Breast Surgery, the First Affiliated Hospital of Xi’an Jiaotong University, Xi’an 710061, China.

^*^Corresponding authors E-mails: [shuangbai@xjtufh.edu.cn](mailto:shuangbai@xjtufh.edu.cn) (S. Bai); luyi169@xjtu.edu.cn (Y. Lyu); gangliu.cmitm@xmu.edu.cn (G. Liu)

^#^Long Liu, Peng Li, and Zhixiang Lu contributed equally to this work.

**Supporting Methods**

**Materials**

Block black phosphorus (BP) was obtained from Nanjing XFNANO Materials (Nanjing, China). Calcium chloride dihydrate (CaCl_2_·2H_2_O), sodium hyaluronate and ammonia solution (NH_3_·H_2_O) were acquired from Aladdin (Shanghai, China). Hydrogen peroxide solution (H_2_O_2_, 30%) was purchased from Merck Life Science (Shanghai, China). The Cell Counting Kit-8 (CCK-8), DCFH-DA, Actin-Tracker Red, Mito-Tracker Green, Lyso-Tracker Green, Fluo-3 AM Ca²⁺ fluorescent probe, JC-1 mitochondrial membrane potential assay kit, Alizarin Red S staining solution, and the One-Step TUNEL apoptosis assay kit were all supplied by Beyotime Biotechnology (Shanghai, China). The MCOLN2 antibody (GTX16616) was purchased from GeneTex Biotechnology (California, USA). Recombinant mouse IL-4 protein (ab259406) used for inducing macrophage polarization was obtained from Abcam (Cambridge, UK). Fluorescently labeled antibodies, including FITC anti-mouse F4/80, FITC anti-mouse CD80, PE anti-mouse CD86, PE anti-mouse CD206, PE anti-mouse CD4, and FITC anti-mouse CD8 were all sourced from Biolegend (Beijing, China).

**Characterization**

The size and micromorphology of BP-CaO_2_ was investigated by transmission electron microscopy (TEM, Tecnai G2 Spirit BioTwin, 120 kV, Japan). The crystal structure and element distribution of BP-CaO_2_ was characterized by High-resolution transmission electron microscopy (HRTEM, JEM-2100F, Japan). The size and thickness of BP-CaO₂ was measured using atomic force microscope (AFM, Bruker, Germany). Malvern Mastersizer 2000 (Zetasizer Nano ZS90, UK) was used to measure zeta potential. The composition and elemental chemical states were determined by X-ray photoelectron spectroscopy (XPS, Thermo Fisher ESCALAB 250Xi XPS). High resolution spectra of Ca 2p, O 1s, and P 2p were collected, and peak deconvolution was performed to obtain the chemical states and binding energy information of the elements. The structure and chemical bonds of BP-CaO_2_ were determined by Fourier transform infrared spectroscopy (FT-IR, Bruker, Germany) through the sample was mixed with KBr and pressed using a pressure machine. X-ray diffraction (XRD) patterns were acquired using a Rigaku D/MAX-2250V diffractometer. Samples were scanned using an X-ray diffractometer over a 2θ range of 10°-80° to analyze their crystal structure and compare the characteristic diffraction peaks of each component.

**Synthesis of BP nanosheets**

The preparation of BP nanosheets was refer to previous method by using a liquid exfoliation technique. Typically, 1 mg of BP crystal was added to 10 mL of NMP and was further performed sonication under ice bath with a power of 900 W for 12 h (On / Off cycle: 10 s / 5 s). Then the BP nanosheets were obtained after centrifuged at 3000 rpm for 3 min to discard multilayer BP nanosheet. Afterwards, the BP nanosheets were stored at 4 ℃ for further use.

**Synthesis of BP-Ca** **nanosheets**

The BP nanosheets were collected by centrifuging at 14,000 rpm for 10 min and washed with methanol for 2 times. The collected BP NSs were dispersed in 10 mL of N-Methyl-2-pyrrolidone (NMP), and ammonia solution (1M) was added to activate BP NSs. Then the CaCl_2_·2H_2_O (1 mL, 1 M) was dropped to the solution and stirring for another 12 h. The product was centrifuged and washed with water to remove excess unloaded calcium ions, obtaining BP-Ca nanosheets.

**Synthesis of BP-CaO_2_ nanosheets**

The BP-CaO₂ nanosheets were synthesized *via* an *in situ* mineralization method. Prepared BP-Ca nanosheets were dispersed in 10 mL of methanol. 1 mL of deionized water was added into the above system. Subsequently, NH₃·H₂O and H₂O₂ were added dropwise. The reaction was allowed to proceed for 1 h. The final product was collected by centrifugation (11,000 rpm, 10 min) and washed with ethanol for 2 times to remove residual reagents, thereby obtaining BP-CaO₂ nanosheets.

**Synthesis of CaO₂ nanoparticles**

CaO₂ nanoparticles were synthesized using similar method. Typically, the CaCl_2_·2H_2_O aqueous solution (2 mL, 1 M) was added into 50 mL of vigorously stirred methanol and stirred for 10 min to obtain a homogeneous mixture. Subsequently, NH₃·H₂O and H₂O₂ was added into the dispersion. The reaction was allowed to proceed for 1 h until the dispersion turned Cambridge blue. The product was collected by centrifugation (11,000 rpm, 10 min) and washed with ethanol for 2 times. The final product of CaO₂ nanoparticles was dried for subsequent experiment.

**Synthesis of CaO₂@HA (CaM) and BP-CaO₂@HA (CaIM)**

To enhance the biocompatibility and stability of BP-CaO₂ for both *in vitro* and *in vivo* therapeutic applications, hyaluronic acid (HA) was employed as a surface coating to fabricate CaO₂@HA (CaM) and BP-CaO₂@HA (CaIM) nanocomposites. Briefly, 1 mg of CaO₂ or 1 mg of BP-CaO₂ was dispersed in 10 mL of anhydrous ethanol to obtain a homogeneous suspension. Subsequently, HA solution was added into the above dispersion. The mixture was then stirred overnight. Then the product was collected by centrifugation and washed with ethanol for 2 times. The final products were obtained as CaM or CaIM for subsequent experiments.

**Determination of drug loading**

The drug loading of calcium peroxide (CaO₂) in the BP-CaO₂@HA nanoparticles (NPs) was quantified using a potassium permanganate (KMnO₄) titration method. In the experiment, a certain amount of BP-CaO₂ was dispersed in DI water, then hydrochloric acid and manganese sulfate were added to the suspension with constant stirring. The resulting mixture was then titrated with a standard 0.02 mol/L KMnO₄ solution until a faint pink color persisted for at least 30 seconds, indicating the endpoint. The drug loading (DL) was calculated according to the following equation:

$$DL\%\left( CaO_{2} \right)=\frac{2.5*c\left( \mathrm{KMn}O_{4} \right)*V\left( \mathrm{KMn}O_{4} \right)*M\left( \mathrm{Ca}O_{2} \right)}{m\left( \mathrm{NP}s \right)}*100\%$$

where c(KMnO₄) and V(KMnO₄) represent the concentration and consumed volume of the KMnO₄ titrant, respectively; M(CaO₂) is the molar mass of calcium peroxide, and m (NPs) is the initial mass of the nanoparticles.

**Ca^2+^ Released from CaIM**

The pH-responsive release of Ca²⁺ was evaluated by dialysis method. 1 mg of CaIM was dispersed in 2 mL of calcium-free buffer solution with different pH values (7.4, 6.8, and 5.0). At scheduled time points, the supernatant was collected by centrifugation and determined by ICP-OES. The cumulative release profiles were subsequently plotted based on the calculated release percentage at each time point to assess the release kinetics under different acidic environments.

**H_2_O_2_ Released from CaIM**

The DCFH probe was used to monitor the generation of H_2_O_2_. The CaIM was added to phosphate buffer with agitation. At designated time points, 100 µL of the release solution was transferred to a 96-well plate and incubated with 100 µL of the activated DCFH probe. The fluorescence intensity was promptly recorded on a microplate reader at an emission wavelength of 525 nm following excitation at 488 nm.

**Cell Lines and Animals**

Mice liver cancer cell line (Hepa1-6), Mouse monocyte-macrophage leukemia cell line (RAW 264.7), and mice liver cancer cell line (H22) were purchased from the Cell Bank of the Chinese Academy of Science. BALB/c female mice (6-8 weeks, 18-22 g) were acquired from the Laboratory Animal Center of Xi’an Jiaotong University (Xi’an, China). All animal experiments were performed in accordance with the protocols approved by the Administrative Committee on Animal Research of Xi’an Jiaotong University.

***In vitro* cellular uptake**

Hepa1-6 cells were plated in 6-well plates and allowed to adhere for 12 h. The culture medium was then replaced with fresh medium containing CaIM, followed by incubation for predetermined time intervals (1 h, 2 h, 4 h, 6 h). Then the cells were washed with PBS and collected. Finally, the quantify cellular uptake was analyzed by flow cytometer. Meanwhile, confocal imaging was used for the intuitive observation of the cellular uptake process. Typically, cells were seeded in confocal dishes and incubated overnight to achieve adherence. CaIM was added and further incubated for 2 h and 6 h. Following incubation, the cells were washed with PBS, fixed with 4% paraformaldehyde, and counterstained with DAPI to visualize the nuclei. The cellular internalization process was then observed by confocal laser scanning microscopy (CLSM).

**Intracellular observation of the release of Ca^2+^ and H_2_O_2_.**

To visualize intracellular Ca²⁺, cells were cultured in confocal dishes and allowed to adhere for 12 h, subsequently treated with control, BP, CaM, and CaIM. After incubation, PBS was used to wash the cells and the intracellular calcium ions were staining by Fluo-3 AM fluorescent probe for 30 minutes at 37 °C. The intracellular fluorescence was then observed under a confocal laser scanning microscope (CLSM).

To assess the generation of intracellular H_2_O_2_, cells were seeded into 12-well plates and cultured for 12 h to ensure attachment. Using control, BP, CaM or CaIM treated cells 6 h and then washed with PBS. DCFH-DA probe was performed to stain cells 30 min and fluorescence intensity was evaluated using a fluorescence microscope to present intracellular H_2_O_2_ level.

***In vitro* cytotoxicity studies**

Cell viability was evaluated using a Cell Counting Kit-8 (CCK-8) assay. Cells were seeded in 96-well plates (10^4^ cells/well) with incubation for 12 h and then treated with control, BP, CaM or CaIM for 24 h. Each well was added 10 µL of CCK-8 solution and cells were incubated for 2 h at 37 °C. Cell viability was determined by measuring the absorbance at 450 nm.

Apoptosis-mediated cell death was quantified using an Annexin V-FITC/Propidium Iodide (PI) Apoptosis Detection Kit. Cells were seeded in 6-well plates and incubated for 12 h. The medium was then replaced with fresh medium containing different formulations (control, BP, CaM, and CaIM) for 24 h. Cell suspension was stained with Annexin V-FITC and PI. Flow cytometry was performed to determine the percentage of apoptotic cells.

Another cell invasion experiments were performed to present more intuitively the inhibitory effect of the CaIM on tumor cells and its possible anti-metastasis function. Cells were seeded in 6-well plates and grown to approximately 90% confluency. A sterile pipette tip was used to create a linear scratch across the center of the cell monolayer. PBS was used to remove detached cells and treated with different formulations. Inverted microscope was utilized to acquire images at 0 h and 24 h. The wound closure area was quantified using ImageJ software.

**Intracellular co-localization assay**

For lysosomal co-localization, cells were seeded into confocal dishes (1 × 10⁵ cells/dish) and allowed to adhere overnight. Following this, the cells were incubated with fresh medium containing the ICG-labeled CaIM for 1 h, 3 h, 6 h, and 9 h. Then the medium was removed, and the cells were washed with PBS. Cells were stained with LysoTracker Green (Lyso-Green) for 30 minutes at 37 °C to visualize the co-localization of lysosomes.

For mitochondrial co-localization, cells were similarly treated with the ICG-labeled CaIM formulations for 6 h and 12 h. Cells were subsequently stained with MitoTracker Green (MT-Green) for 30 minutes at 37 °C to visualize the mitochondria. After staining for either organelle, cells were washed with PBS, and the intracellular fluorescence was immediately observed using a CLSM. The red fluorescence channel was used to capture the ICG-labeled CaIM, the green channel was used for the respective organelle trackers (MT-Green or Lyso-Green), and the merged images were analyzed to assess co-localization.

**Investigation of Mitochondrial dysfunction**

Mitochondrial membrane potential was assessed using a JC-1 fluorescent probe. Cells were seeded in confocal dishes at a density of 5 × 10⁴ cells per well. The culture medium was subsequently replaced by fresh DMEM supplemented with CaIM, followed by incubation for 6 h. After treatment, the cells were gently washed with PBS and stained with the JC-1 probe according to the manufacturer’s instructions. Membrane potential change was monitored using confocal laser scanning microscope (CLSM).

Bio-transmission electron microscopy (Bio-TEM) imaging was employed to observe the ultrastructure of subcellular organelles. Cells were incubated with control, BP, CaM, CaIM for 6 h. Then the cells were harvested and fixed with glutaraldehyde, post-fixed with osmium tetroxide, dehydrated through a graded ethanol series, and embedded in epoxy resin. Ultrathin sections were then prepared and stained with uranyl acetate and lead citrate for analysis by Bio-TEM.

***In vitro* cell calcification detection**

A series of assays were performed on tumor cells to comprehensively evaluate intracellular Ca²⁺ and biological calcification, including bio-transmission electron microscopy (Bio-TEM), scanning electron microscopy (SEM), and alizarin red staining. Cells were seeded into a 6-well plate and cultured overnight. After that, the cells were treated with fresh medium containing different formulations (control, BP, CaM, and CaIM) for 6 h. Then, the cells were collected to successively conduct dehydrated, embedded, sliced, and stained processed. The ultrastructure of calcified vesicles was evaluated by Bio-TEM.

For elemental analysis, cells were cultured on silicon wafers. After cell adherence, the cells were treated with fresh medium containing different formulations (control, BP, CaM, and CaIM) for 24 h. Then, the cells were successively conduct fixed and dehydrated processed. The images were obtained by scanning electron microscopy (SEM), and the elemental composition of O, Ca and P was analyzed using energy-dispersive X-ray spectroscopy (EDS).

Cellular calcification was further confirmed by Alizarin Red staining. Cells were seeded into 12-well plate and cultured overnight. After that, the cells were treated with fresh medium containing different formulations (control, BP, CaM, and CaIM) for 24 h. Then the cells were conduct fixed with 95% ethanol for 1 h, washed, and stained with an Alizarin Red S solution for 30 minutes at room temperature. The resulting red mineralized nodules were observed under an optical microscope.

***In vitro*** **macrophage polarization detection**

RAW 264.7 macrophages were seeded in 6-well plates (1 × 10^6^ cells/well). To induce M2 polarization, the cells were treated with DMEM containing 50 ng/mL IL-4 for 24 h. Subsequently, the medium was removed, and the cells were incubated for another 24 h with control, BP, CaM, or CaIM. The cells from each group were harvested and washed with PBS. The cell suspensions were then stained with anti-CD86 and anti-CD206 antibodies. Expression of cell surface markers CD86 and CD206 were detected by flow cytometry to evaluate macrophage polarization state.

**Biological safety study of CaIM**

Hemolysis assay was performed to assess the hemocompatibility of CaIM. Fresh whole blood was collected from healthy mice into EDTA-containing tubes, and red blood cells (RBCs) were isolated by repeated centrifugation and washing with phosphate-buffered saline without calcium and magnesium. CaIM dispersions at different concentrations (final concentrations: 10, 20, 40, 100, 200, 400, and 800 μg/mL) were incubated with the RBCs (4%) suspension at 37 ℃ for 3 h. RBCs cultured with PBS or H_2_O were served as negative and positive control, respectively. Pictures were taken of samples and the cell hemolysis was tested by UV spectrophotometer. The results of hemolysis were calculated by the following formula:

$$hemolysis rate \left( \% \right)=\frac{sample absorbance-negative control}{positive control-negative control}\times100\%$$

To assess the *in vivo* biosafety of CaIM, healthy mice were randomly assigned into two groups, including control group with saline treated and CaIM group. Mice in each group received a single intravenous injection of saline or CaIM at a dose of 10 mg/kg. At 7 days of administration, blood samples were collected *via* retro-orbital puncture for routine hematological analysis and serum biochemical examinations.

***In vivo* antitumor performance.**

*Subcutaneous tumor model*

To establish the subcutaneous tumor model, approximately 1×10^7^ tumor cells suspended in 100 μL of PBS were subcutaneously implanted into the right hind limb of each mouse. Following implantation, the mice were closely monitored for tumor growth. Tumor volumes were calculated using the formula:

$$Volume\left( V \right)=\frac{length\times{width}^{2}}{2}$$

*In vivo antitumor efficacy*

The tumor-bearing mice were randomly divided into four treatment groups (n=6) when the tumor volumes reached about 80 mm^3^ and. Each mouse received an intratumoral injection of 50 μL (10 mg/kg) of the corresponding formulation (saline, BP, CaM, and CaIM) on 0, 3, and 6 days. Tumor volume and body weights of mice were monitored every 2 days, respectively. Following humane euthanasia on day 12 post-treatment, tumor tissues, major organs, and blood samples were procured for subsequent analysis.

*Flow cytometry analysis of immune cell populations*

Tumor tissues, tumor-draining lymph nodes, and spleens were collected from mice in the control, BP, CaM, and CaIM treatment groups. Fresh samples were homogenized and dissociated into single-cell suspensions. Flow cytometry analysis was performed to evaluate macrophage polarization using anti-CD86 and anti-CD206 antibodies. Dendritic cells maturation was detected and assessed based on anti-CD86 and anti-CD206 antibodies. T cells activation was estimated by staining with anti-CD86 and anti-CD206 antibodies. All data analysis was processing using FlowJo software (Tree Star, USA).

*In vivo tumor calcification detection.*

Small-animal computed tomography (CT) was employed to noninvasively visualize intratumoral mineral deposition. CT scans were performed at 7 days after the last injection using a high-resolution micro-CT imaging system. Transverse images were acquired to evaluate radiodensity changes within tumor tissues. Quantitative analysis of CT values was conducted by measuring relative radiodensity within tumor regions. Sagittal and coronal CT images was reconstructed to assess spatial distribution and continuity of tumor biomineralization. Three-dimensional (3D) CT reconstruction was subsequently performed to visualize the overall morphology of tumor calcification *in vivo*.

Additionally, isolated tumor tissues were removed from mice and rotational CT imaging (0°-360°) was conducted to confirm the spatial stability and continuity of calcified regions. Continuous slice-by-slice CT analysis was performed across tumor sections to evaluate the distribution of mineral deposits throughout the tumor volume.

Calcium salt deposition was further evaluated using Alizarin Red staining. Following *in vivo* and *ex vivo* CT imaging, tumor tissues were fixed, paraffin-embedded, and sectioned. The sections were deparaffinized, rehydrated, and stained with Alizarin Red S solution for 30 min at room temperature. The optical microscope was used to observe stained sections, evaluating the distribution of calcium salt deposition among different treatment groups.

To further verify the elemental composition of the deposited minerals, scanning electron microscopy (SEM) coupled with energy-dispersive X-ray spectroscopy (EDS) was employed. Tumor samples were dehydrated, dried, and sputter-coated with gold prior to SEM observation. Elemental mapping and quantitative analysis of carbon (C), oxygen (O), phosphorus (P), and calcium (Ca) were performed using EDS to determine the composition and spatial distribution of mineral deposits.

*Histological staining and* *imaging assessment*

To evaluate therapeutic efficacy and biological safety, tumor tissues and major organs of mice were harvested after treatment. All samples were fixed in 4% formalin, dehydrated, embedded in paraffin, and serially sectioned. For general morphological evaluation and systemic toxicity assessment, Hematoxylin and Eosin (H&E) was performed on tumors and major organs, including heart, liver, spleen, lung, and kidney. To evaluate anti-tumor effects, tumor sections were subjected to a series of specific staining procedures. Apoptotic cells were detected using a TUNEL assay. Proliferation was assessed by immunostaining for Ki67. Tumor calcification was visualized using Alizarin Red staining. Immunofluorescence staining was performed for various markers, including CD80, CD86, CD206, CD4, and CD8.

**Western blotting**

Total proteins were extracted using RIPA buffer supplemented with protease and phosphatase inhibitor cocktails. 25 µg of proteins were separated by 10% SDS-PAGE gels and transferred to PVDF membranes. Using a rapid blocking solution to block proteins for 20 min at room temperature and indicated primary antibodies to incubate proteins at 4 ℃ overnight. HRP-conjugated secondary antibodies were further incubated at room temperature and chemiluminescent signals were visualized by chemiluminescence imaging system (Tanon, Shanghai, China). MCOLN2 (1:1000, GeneTex, USA), β-Actin (1:10000, Abcam, UK) were used as primary antibodies.

**Proteomic and phosphoproteomic profiling**

Protein extraction and digestion from tumor tissues were performed using the Filter-Aided Sample Preparation (FASP) approach to ensure efficient removal of contaminants and high peptide recovery. The resulting peptides were chemically labeled with TMT10plex reagents (Pierce, Thermo Fisher Scientific, USA) following the manufacturer’s instructions for multiplexed quantification. Approximately 1.8 mg of labeled peptides were fractionated by basic reversed-phase (bRP) chromatography on an Ultimate 3000 HPLC system (Dionex, USA) equipped with a Waters XBridge BEH C18 column (5 μm, 4.6 × 250 mm). Peptides were eluted with a linear gradient, and the collected fractions were lyophilized for subsequent analysis. For LC–MS/MS acquisition, samples were reconstituted in 0.1% formic acid (FA) and analyzed on an Orbitrap Fusion Lumos mass spectrometer (Thermo Fisher Scientific). Peptide separation was achieved on a C18 analytical column (75 μm × 25 cm, 2 μm particle size) using a linear gradient elution. The instrument operated under data-dependent acquisition (DDA) mode to automatically select precursor ions for fragmentation. Raw spectra were processed with Proteome Discoverer software (version 2.4), and database searches were performed against the UniProt Oryctolagus cuniculus reference proteome (UP000001811). Search parameters included a precursor ion tolerance of 20 ppm and a fragment ion tolerance of 0.02 Da. Peptide and protein identifications were filtered to maintain a false discovery rate (FDR) below 1%, with at least one unique peptide required for confident protein assignment.

**Proteomic analysis**

The quantitative proteomic data obtained from TMT-based LC-MS/MS were log2-transformed and normalized using the normalizeBetweenArrays function. Principal component analysis (PCA) was performed using the *scatterplot3d* R packages to visualize global proteome differences among the control, BP, CaM, and CaIM groups. Differentially expressed proteins between experimental groups were identified using *limma* with P value < 0.05 and | log₂ (fold change) | > 1 as thresholds. Volcano plots were generated using *ggplot2*, and representative proteins were displayed using boxplots based on normalized expression values. Heatmaps exhibiting protein expression patterns across samples were drawn using the *pheatmap* package, with Z-score normalization applied to highlight relative changes. Differentially expressed proteins were uploaded to the STRING database (version 11.5) and further generated protein-protein interaction (PPI) network using a confidence score cutoff of 0.7. Cytoscape (version 3.9.1) was employed to visualize and cluster PPI network. Key subnetworks were further identified using the MCODE plugin. Functional enrichment analysis of GO and KEGG were performed utilizing differentially expressed proteins by *clusterProfiler* package. Gene Set Enrichment Analysis (GSEA) was conducted based on all quantified proteins ranked by fold change to assess coordinated biological alterations.

**Phosphoproteomic analysis**

For phosphoproteomic profiling, phosphorylation of serine, threonine, and tyrosine residues were specified as variable modifications. The proportions of phosphorylated serine (pS), threonine (pT), and tyrosine (pY) residues were calculated from all confidently identified sites and visualized using a proportional pie chart to reflect the overall phosphorylation distribution across the proteome. Sequence motifs surrounding phosphorylation sites were analyzed to uncover potential kinase recognition patterns. For each identified phosphosite, amino acid sequences around the modification site were extracted and analyzed using the Motif-X algorithm. Sequence logos were visualized using *ggseqlogo* R package, highlighting conserved residues adjacent to phosphorylated amino acids.

**Transcriptome profiling**

Transcriptome data were retrieved from four independent GEO datasets, including GSE144269, GSE54236, GSE109211, and GSE173839. Raw expression matrices and corresponding clinical annotations were downloaded using the GEOquery package. Probe identifiers were converted to gene symbols according to the platform annotation files, and for genes with multiple probes, the median expression value was used. Gene expression was normalized by z-score transformation within each dataset. GSE144269 and GSE54236 contained samples from tumor and adjacent normal tissues, while GSE109211 and GSE173839 included patients with sorafenib drug treatment and anti-PD-L1 immunotherapy, respectively. We conducted part of the data analysis using BEST, which is freely accessible to all users without any login requirements. Among distinct datasets, gene expression was visualized using *ggplot2* package. Immune cell infiltration was evaluated by ESTIMATE and xCELL algorithm. Correlation analysis between gene expression and cell infiltration was performed using Pearson correlation coefficient.

**Single-cell** **RNA sequencing**

GSE156625 as a single-cell RNA sequencing data contained patients with hepatocellular carcinoma. Single-cell transcriptomic data were analyzed using the *Seurat* v4.0 pipeline following standard quality control, normalization, and dimensionality reduction procedures. Cells with fewer than 200 detected genes or >20% mitochondrial reads were excluded. Gene expression matrices were normalized using LogNormalize and scaled, with top 2,000 highly variable genes identified via the vst method. Dimensionality reduction was performed using principal component analysis (PCA). Uniform manifold approximation and projection (UMAP) was applied to visualize cell clusters. Based on specific marker genes and CellMarker database (e.g., CD3D for T cells, ALB for hepatocytes), cell clusters were annotated in GSE156625. Characteristic genes of cell subpopulations were identified by FindAllMarkers function. Signatures of M1 macrophage and M2 macrophage were collected and quantized using AUCELL algorithm.

**Supporting Figures:**


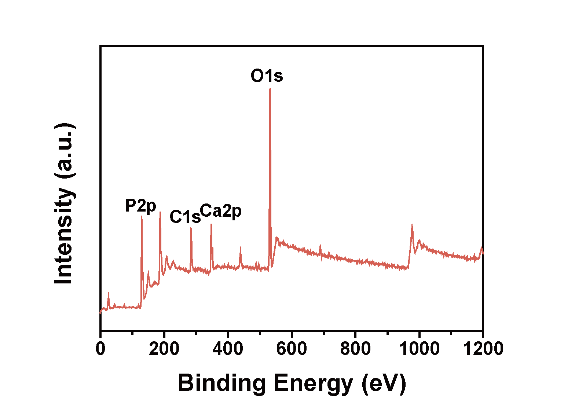


**Figure S1. XPS spectrum of BP-CaO_2_.** XPS survey spectrum showing the characteristic elemental signals, including O 1s, C 1s, Ca 2p, and P 2p peaks across the full binding energy range.


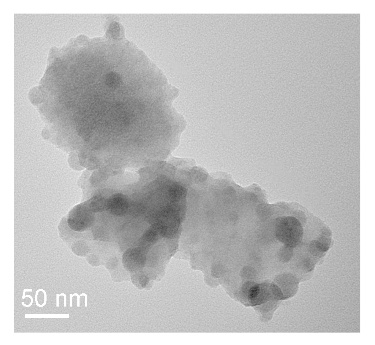


**Figure S2. TEM characterization of CaIM nanoparticles.** Transmission electron microscopy (TEM) images of BP-CaO_2_@HA (CaIM).


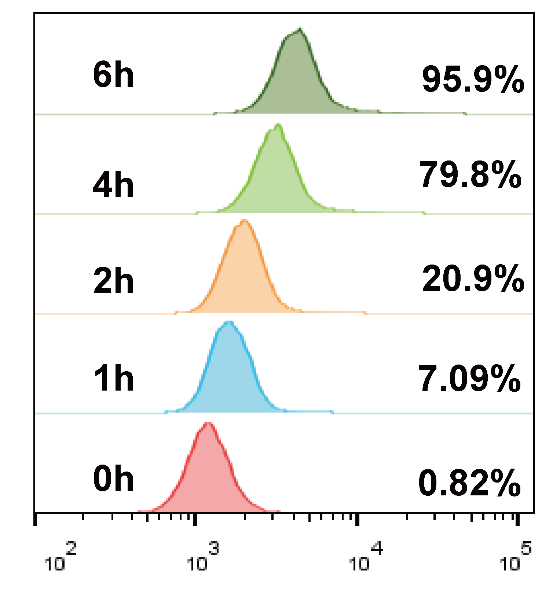


**Figure S3. Time-dependent cellular uptake of CaIM.** Flow cytometry analysis of CaIM uptake by tumor cells over time.


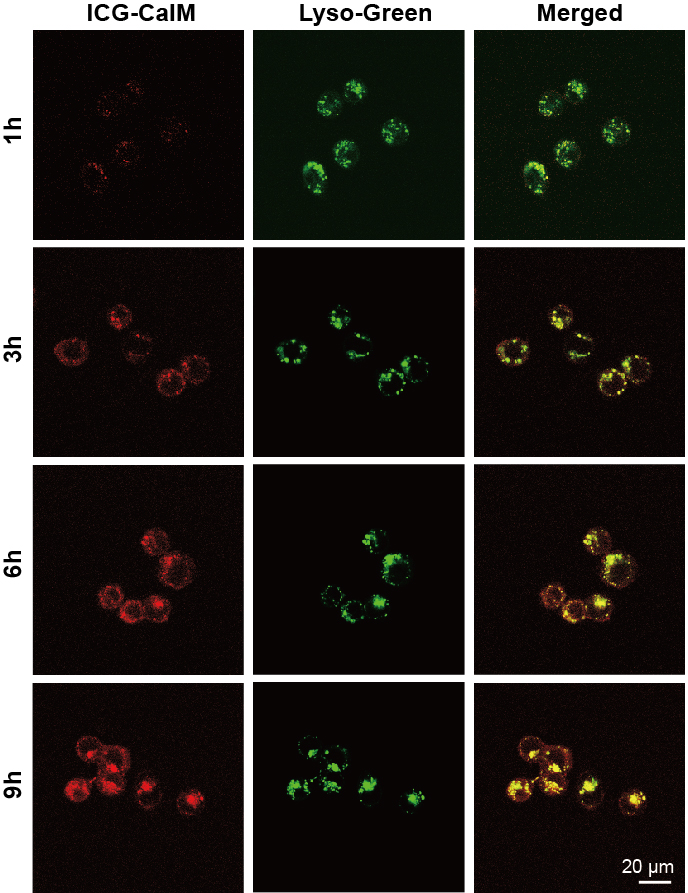


**Figure S4. Lysosomal colocalization and escape of CaIM.** Lyso-tracker staining for lysosome colocalization and lysosomal escape. Confocal fluorescence images showing the intracellular colocalization of CaIM labeled by ICG (red) with lysosomes stained by Lyso-Green (green), with merged channels indicating overlap of signals (scale bar: 20 μm).


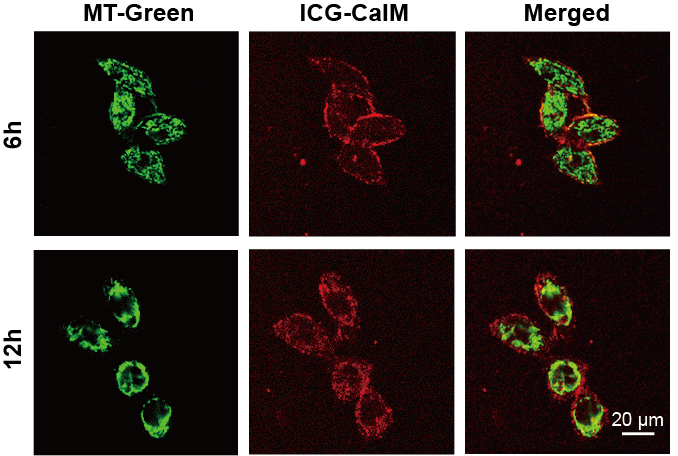


**Figure S5. Mitochondrial colocalization of CaIM.** Mito-tracker staining for mitochondria colocalization. Confocal fluorescence images showing the colocalization of CaIM labeled by ICG (red) with mitochondria labeled by MT-Green (green), with merged channels indicating overlap of signals (scale bar: 20 μm).


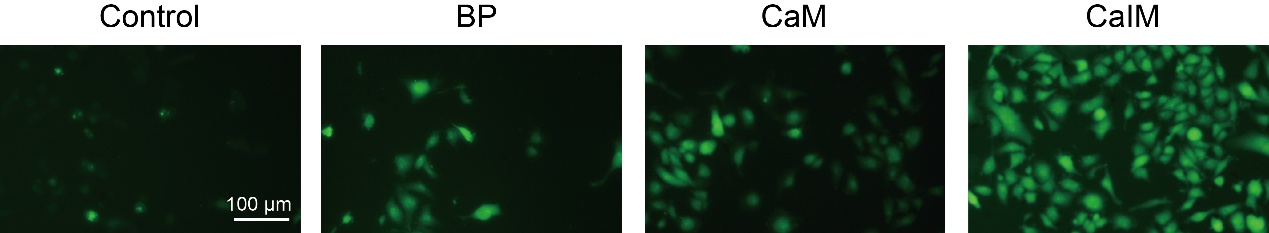


**Figure S6. Intracellular H₂O₂ generation by CaIM.** DCFH-DA fluorescent probe was used to detect the H_2_O_2_ production (scale bar: 100 μm).


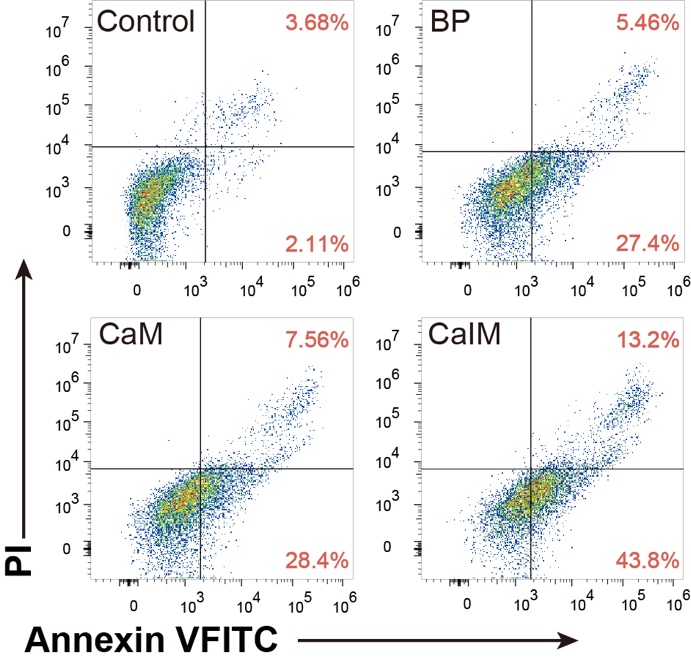


**Figure S7**. **CaIM-induced apoptosis assessed by flow cytometry.** Flow cytometry analysis of apoptosis using Annexin V-FITC/PI staining.


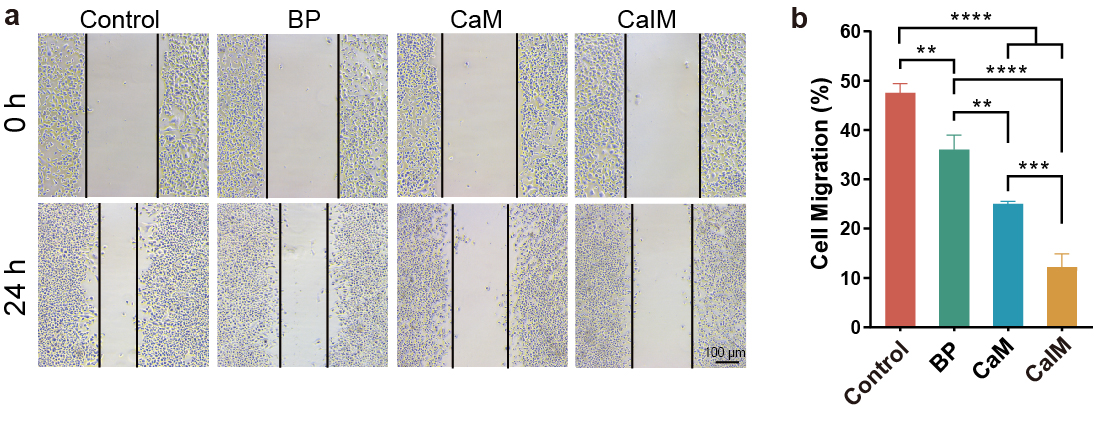


**Figure S8. CaIM inhibits tumor cell migration.** a) Representative images of the cell migration assay in control, BP, CaM, and CaIM groups, assessing cell migration (scale bar: 100 μm). b) Quantification of cell migration assays for treated tumor cells.


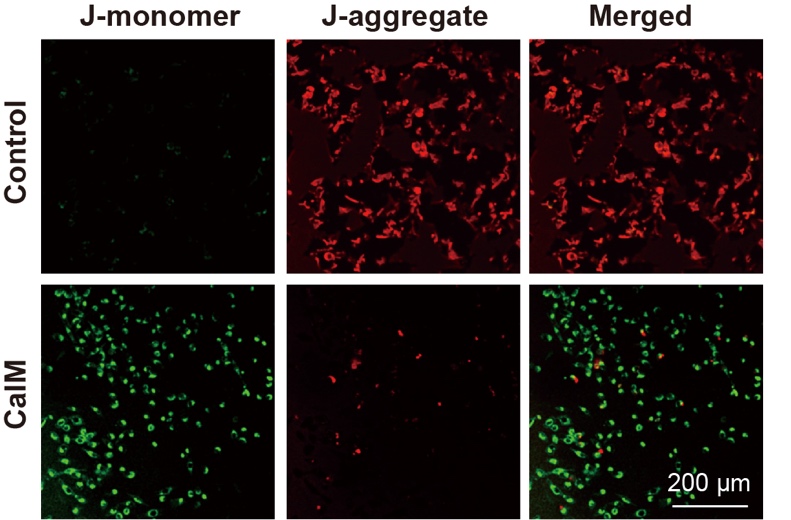


**Figure S9. CaIM disrupts mitochondrial membrane potential.** JC-1 staining displaying mitochondrial membrane potential changes (scale bar: 200 μm).


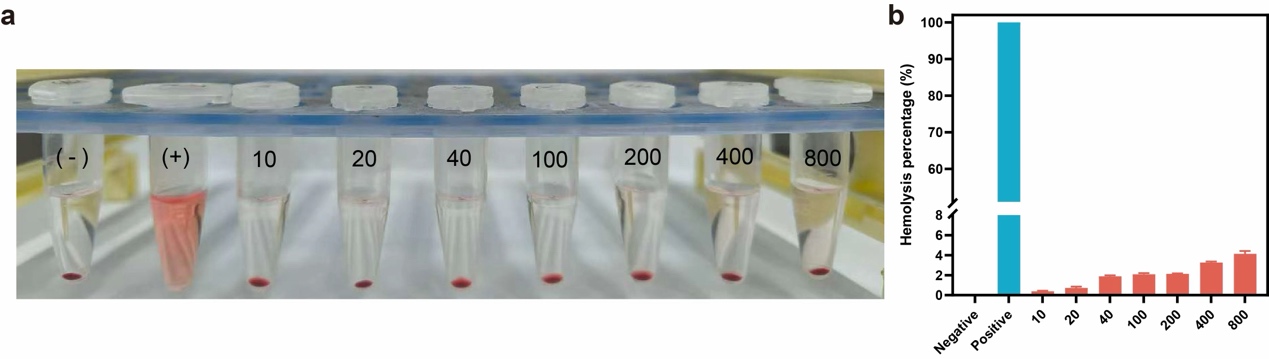


**Figure S10. Hemocompatibility evaluation of CaIM.** a) Photographs of the hemolysis assay displaying red blood cell suspensions treated with negative control (–), positive control (+), and the CaIM at concentrations of 10, 20, 40, 100, 200, 400, and 800 μg/mL. b) Quantitative analysis of hemolysis percentage determined by UV absorption at 540 nm.


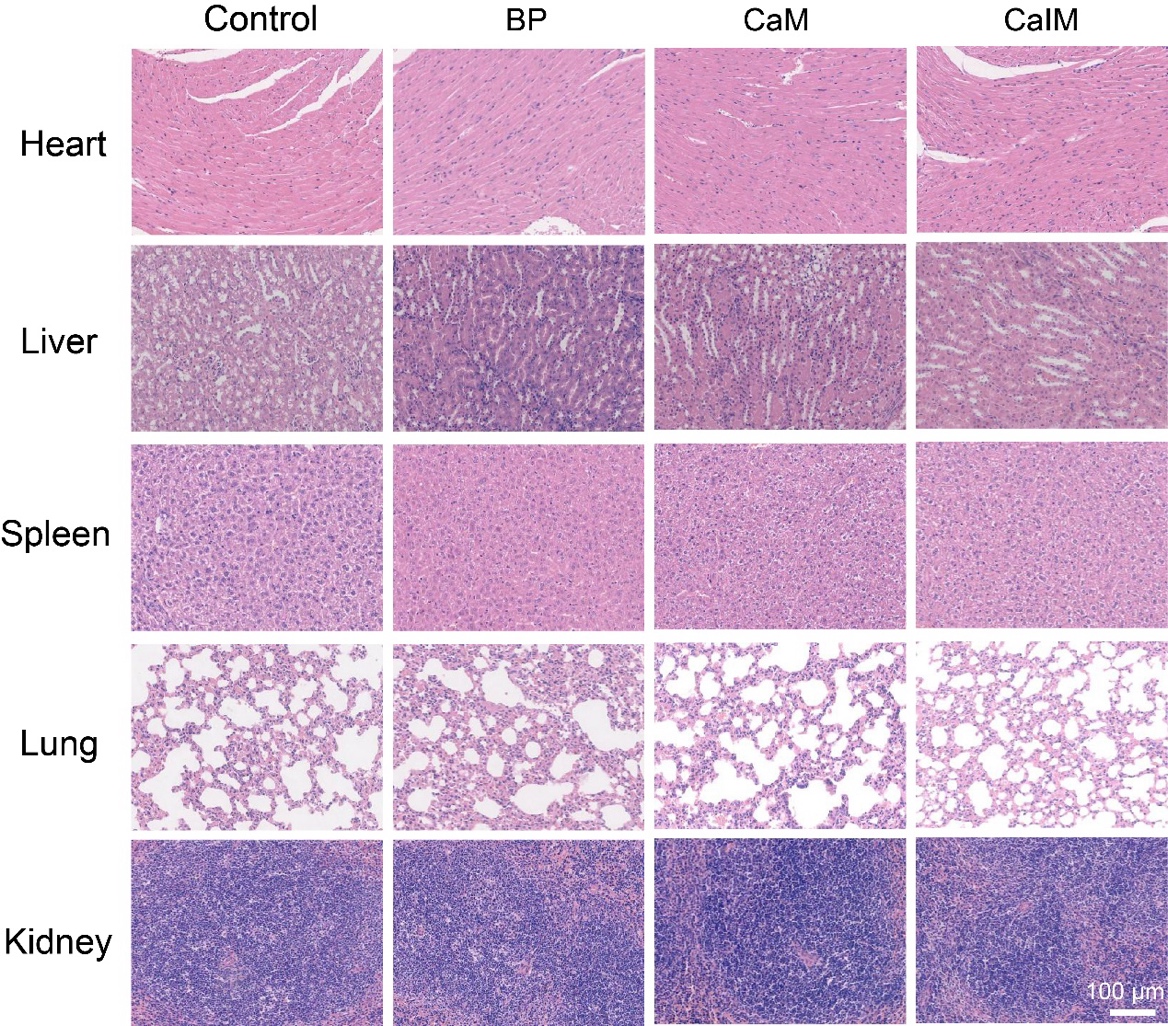


**Figure S11. Histological assessment of organ safety.** H&E staining images of major organs (heart, liver, spleen, lung and kidney) of subcutaneous tumor mouse model after different treatments. Images are representative data from one of six independent mice in each group (scale bar: 100 μm).


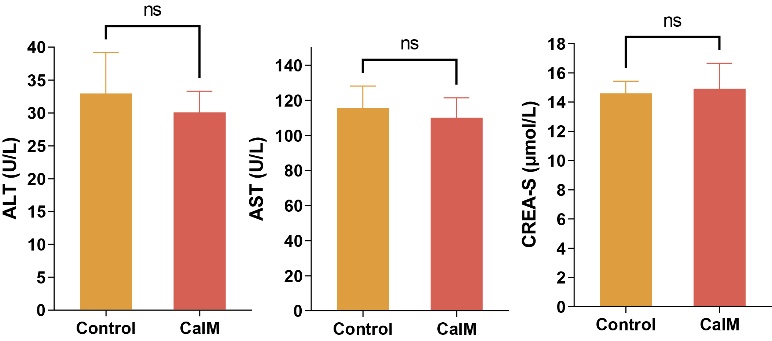


**Figure S12. Serum biochemistry safety analysis.** Blood biochemistry analysis after different treatments. Indicators included alanine aminotransferase (ALT), aminotransferase (AST), serum creatinine (CREA-S).


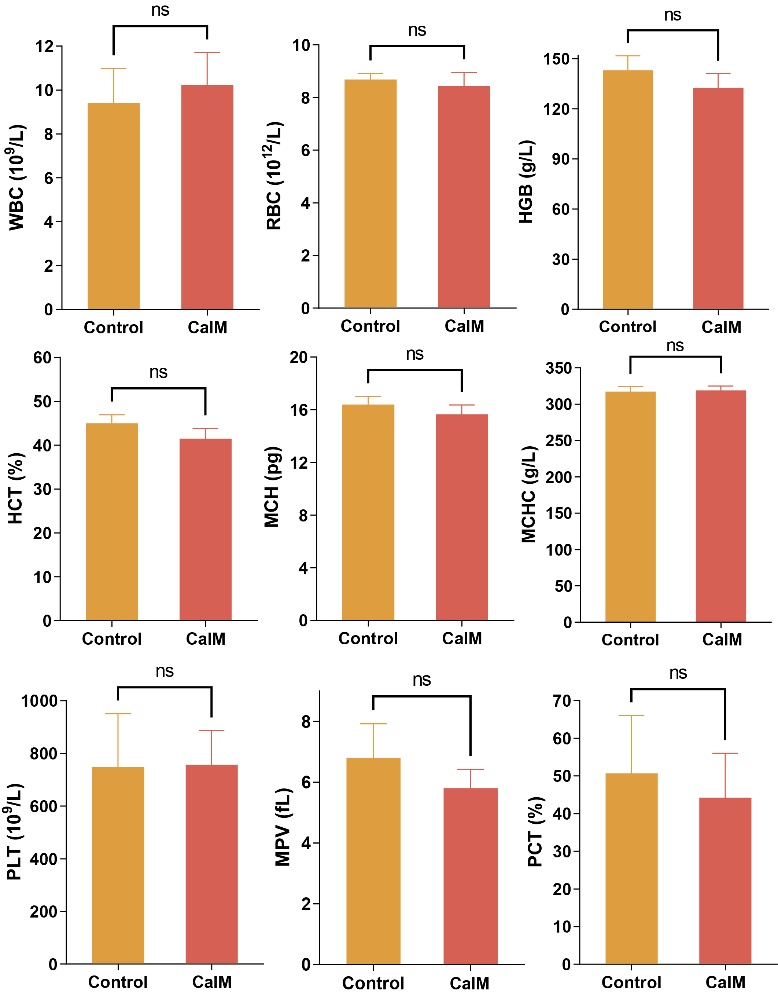


**Figure S13. Hematological safety analysis.** Blood routine analysis after different treatments. Indicators included white blood cell (WBC), red blood cell (RBC), hemoglobin (HGB), hematocrit (HCT), mean corpuscular hemoglobin (MCH), mean corpuscular hemoglobin concentration (MCHC), blood platelet (PLT), mean platelet volume (MPV), and Plateletcrit (PCT).


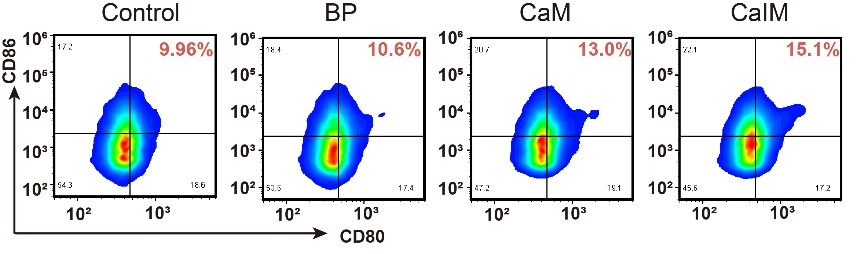


**Figure S14. Dendritic cell maturation in the spleen by flow cytometry.** Representative plots of CD86 and CD80 staining of dendritic cells in the spleen tissue measured by flow cytometry.


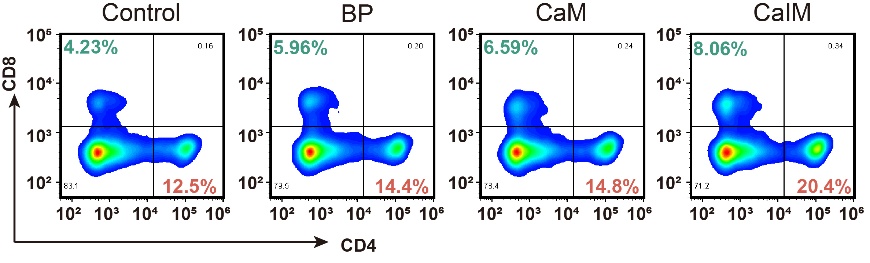


**Figure S15. T cell populations in the spleen by flow cytometry.** Representative plots of CD4 and CD8 staining of T cells in the spleen tissue measured by flow cytometry.


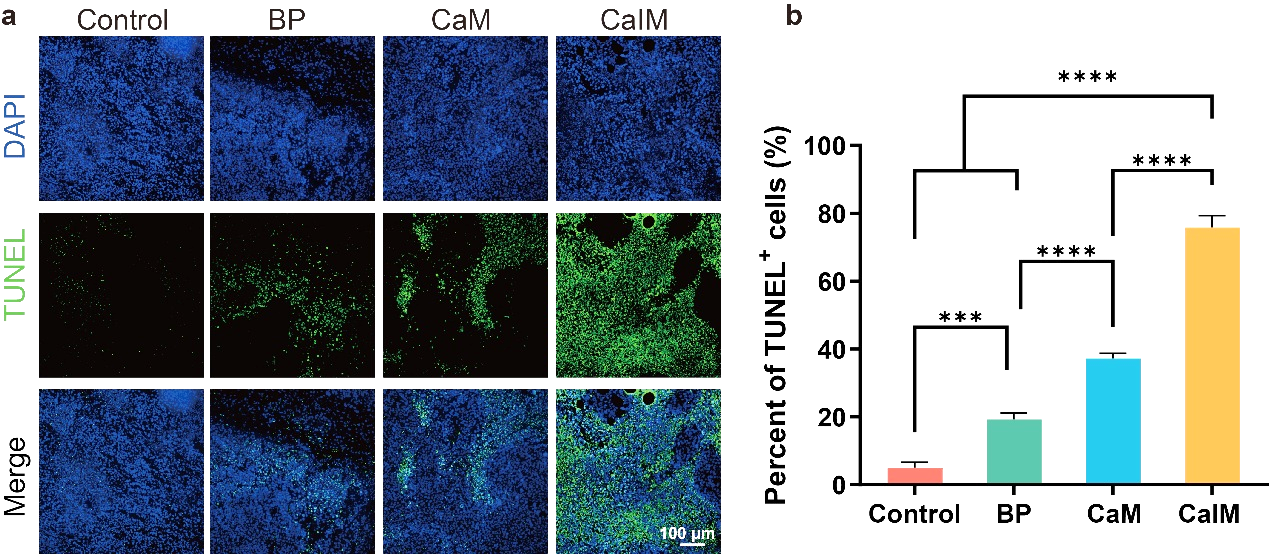


**Figure S16. TUNEL staining for tumor apoptosis.** a) TUNEL staining of the tumor tissues with different treatments. b) Quantitative analysis of TUNEL staining of the tumors. Scale bar: 100 μm.


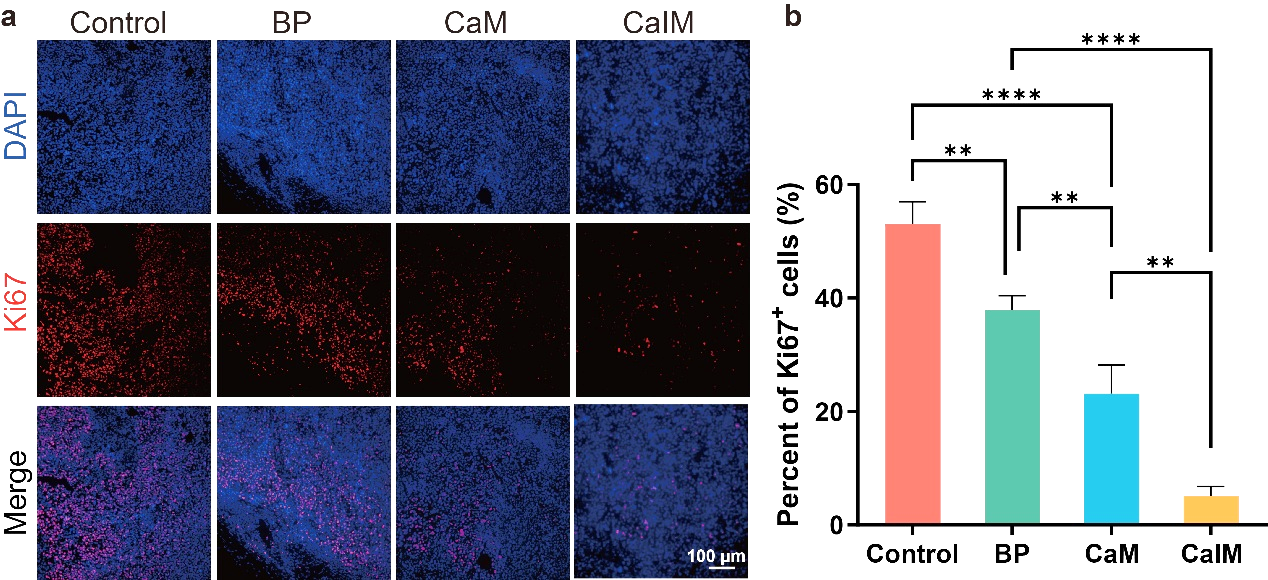


**Figure S17. Ki67 staining for tumor proliferation.** a) Ki67 staining of the tumor tissues with different treatments. b) Quantitative analysis of Ki67 staining of the tumors. Scale bar: 100 μm.


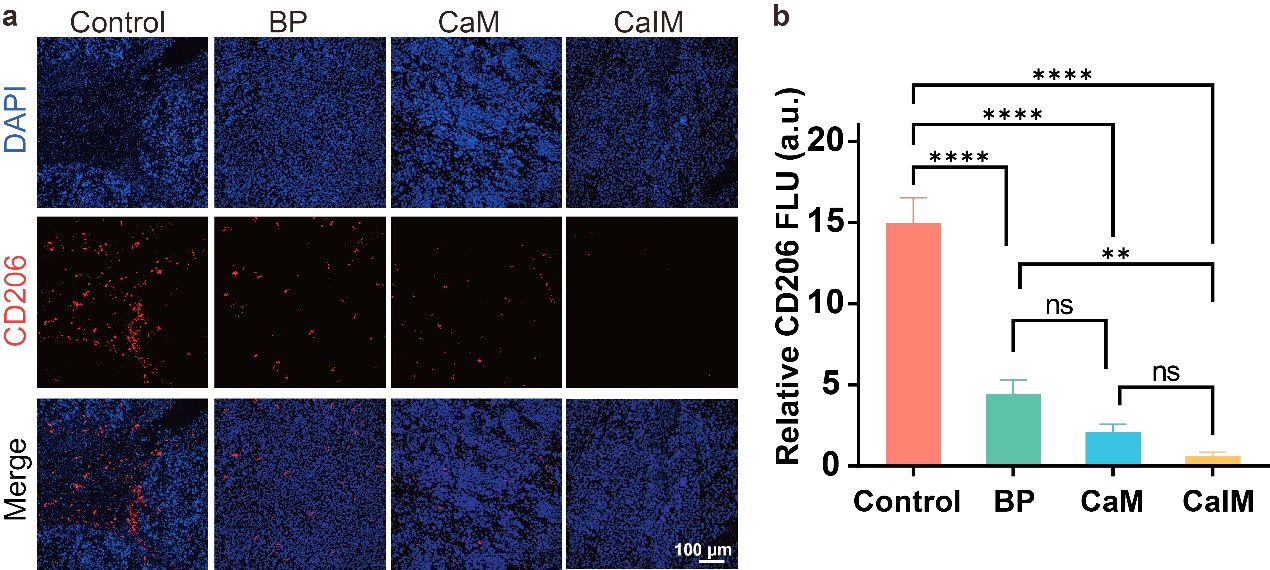


**Figure S18. M2 macrophage polarization in tumor tissues.** a) Immunofluorescence image of CD206 in the tumor tissues after different treatments. b) Quantitative immunofluorescence analysis of CD206 in the tumor tissues after different treatments. Scale bar: 100 μm.

**
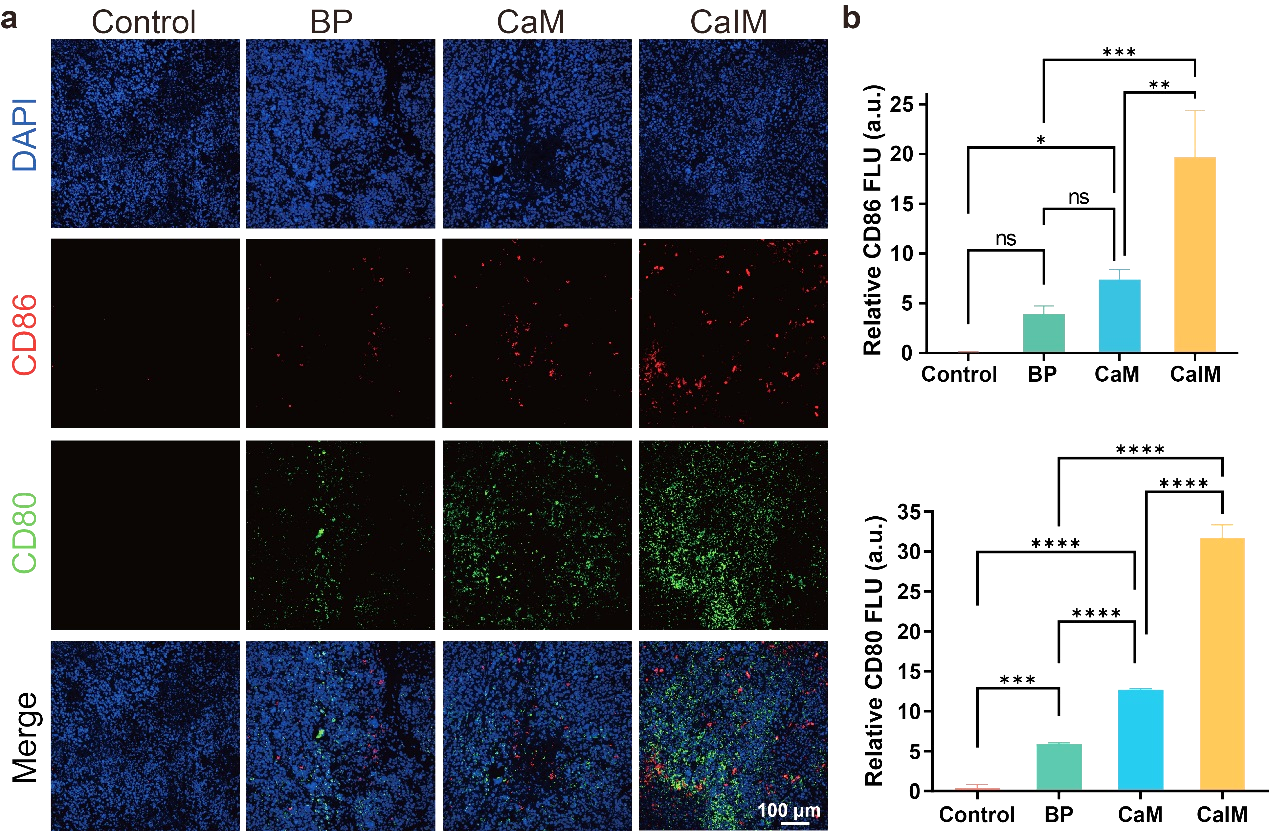
**

**Figure S19. Dendritic cell activation in tumor tissues.** a) Immunofluorescence image of CD86 and CD80 in the tumor tissues after different treatments. b) Quantitative immunofluorescence analysis of CD86 and CD80 in the tumor tissues after different treatments. Scale bar: 100 μm.

**
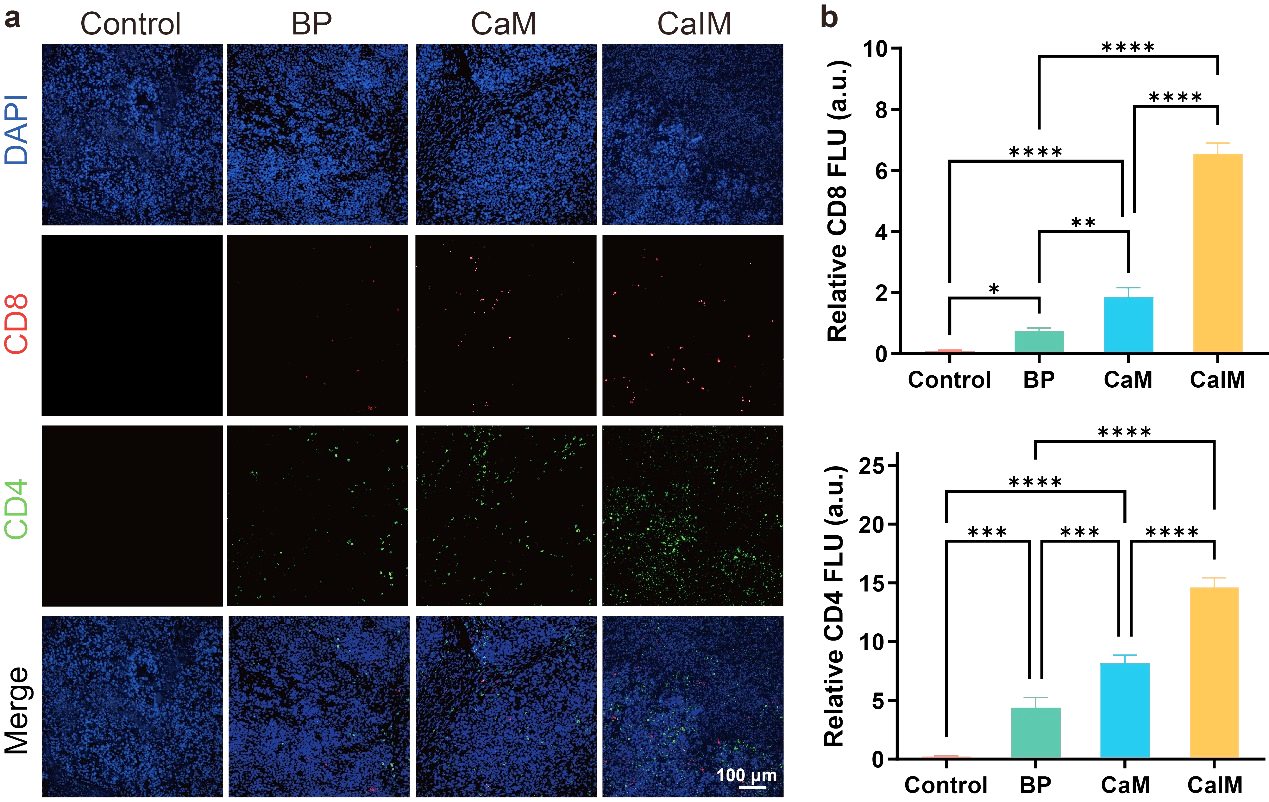
**

**Figure S20. T cell infiltration in tumor tissues.** a) Immunofluorescence image of CD8 and CD4 in the tumor tissues after different treatments. b) Quantitative immunofluorescence analysis of CD8 and CD4 in the tumor tissues after different treatments. Scale bar: 100 μm.


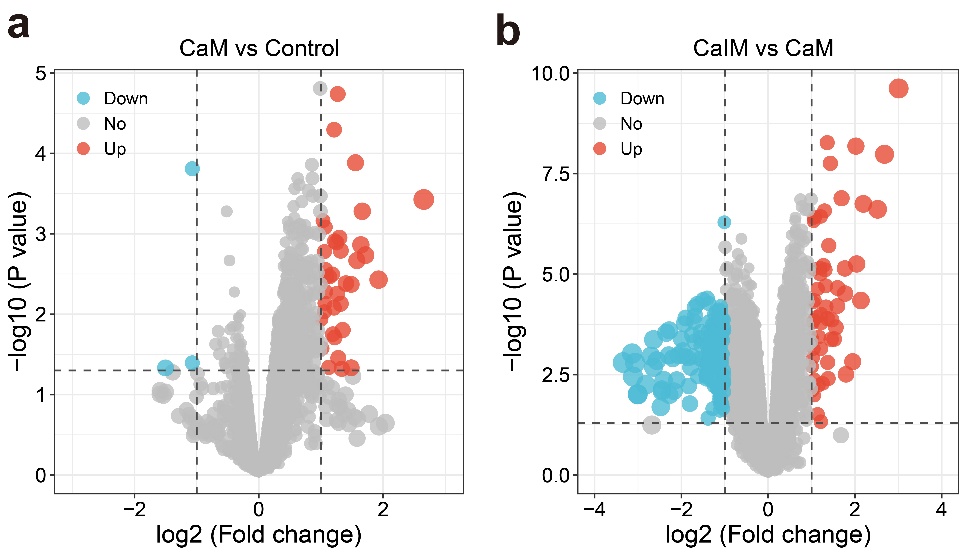


**Figure S21. Differentially expressed proteins identified by volcano plots.** a) Volcano plot showing differentially expressed proteins between the CaM and control groups. b) Similarly, differentially expressed proteins between the CaIM and CaM groups, with upregulated (red), downregulated (blue), and non-significant (gray) proteins indicated.


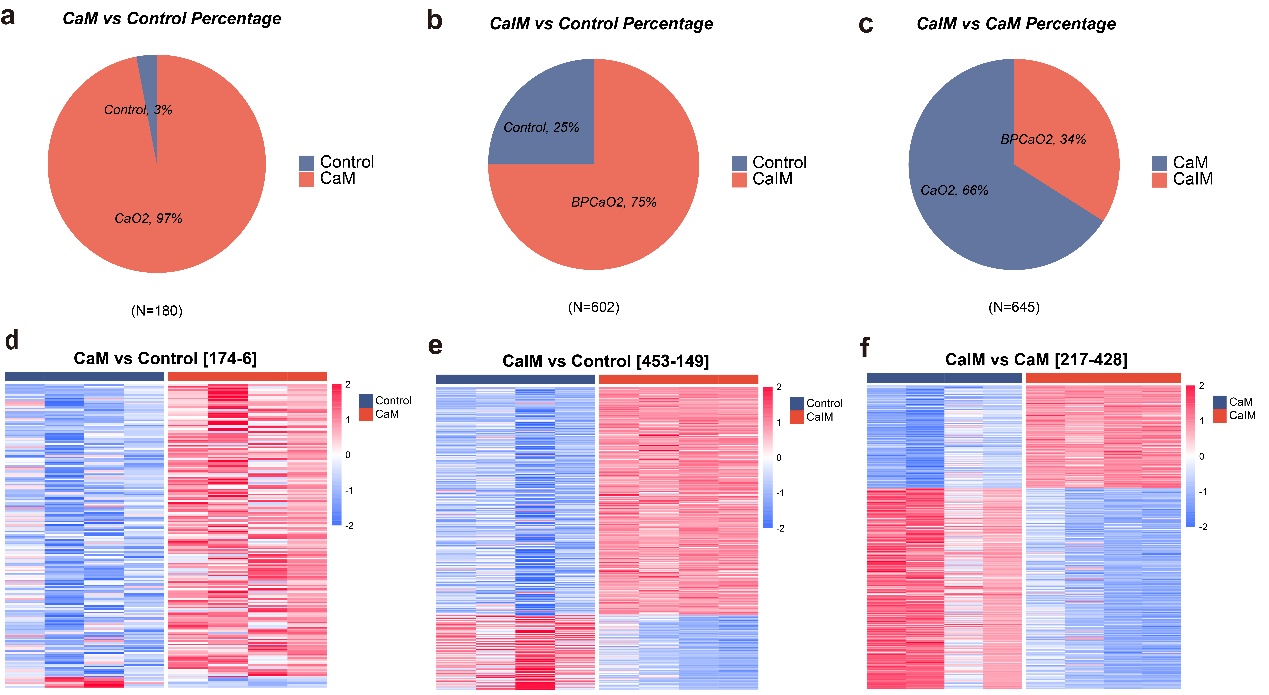


**Figure S22. Proteomic profiling across treatment groups.** Differential protein distribution and expression profiles among treatment groups based on proteomics data. a-c) Pie chart showing the up-regulated or down-regulated proteins in CaM and control groups (a), CaIM and control groups (b), and CaIM and CaM groups (c), respectively. d-f) Heatmap of the up-regulated or down-regulated proteins in CaM and control groups (d), CaIM and control groups (e), and CaIM and CaM groups (f), respectively.


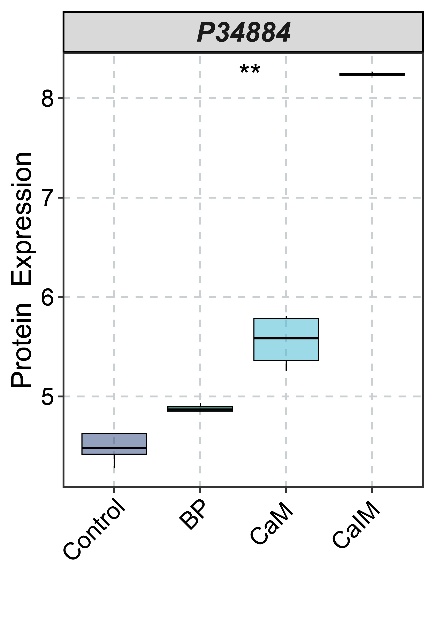


**Figure S23. Expression levels of protein P34884.** Box plot displaying the expression levels of protein P34884 in control, BP, CaM, and CaIM groups.


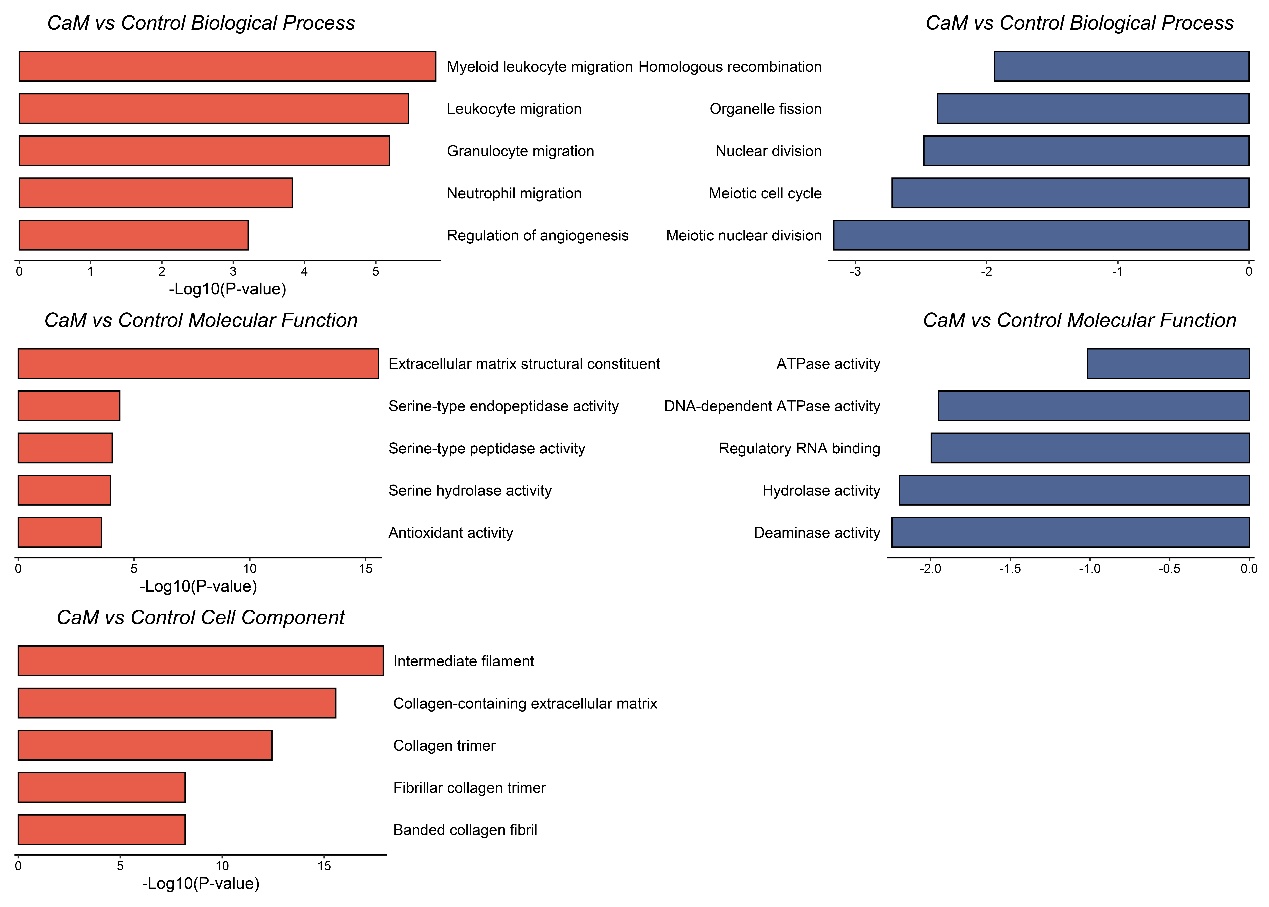


**Figure S24. GO enrichment analysis of CaM-regulated proteins.** GO enrichment analysis between CaM and control groups in “Biological Process”, “Molecular Function”, and “Cellular Component” gene terms. Genes were enrolled using up-regulated or down-regulated proteins between CaM and control groups.


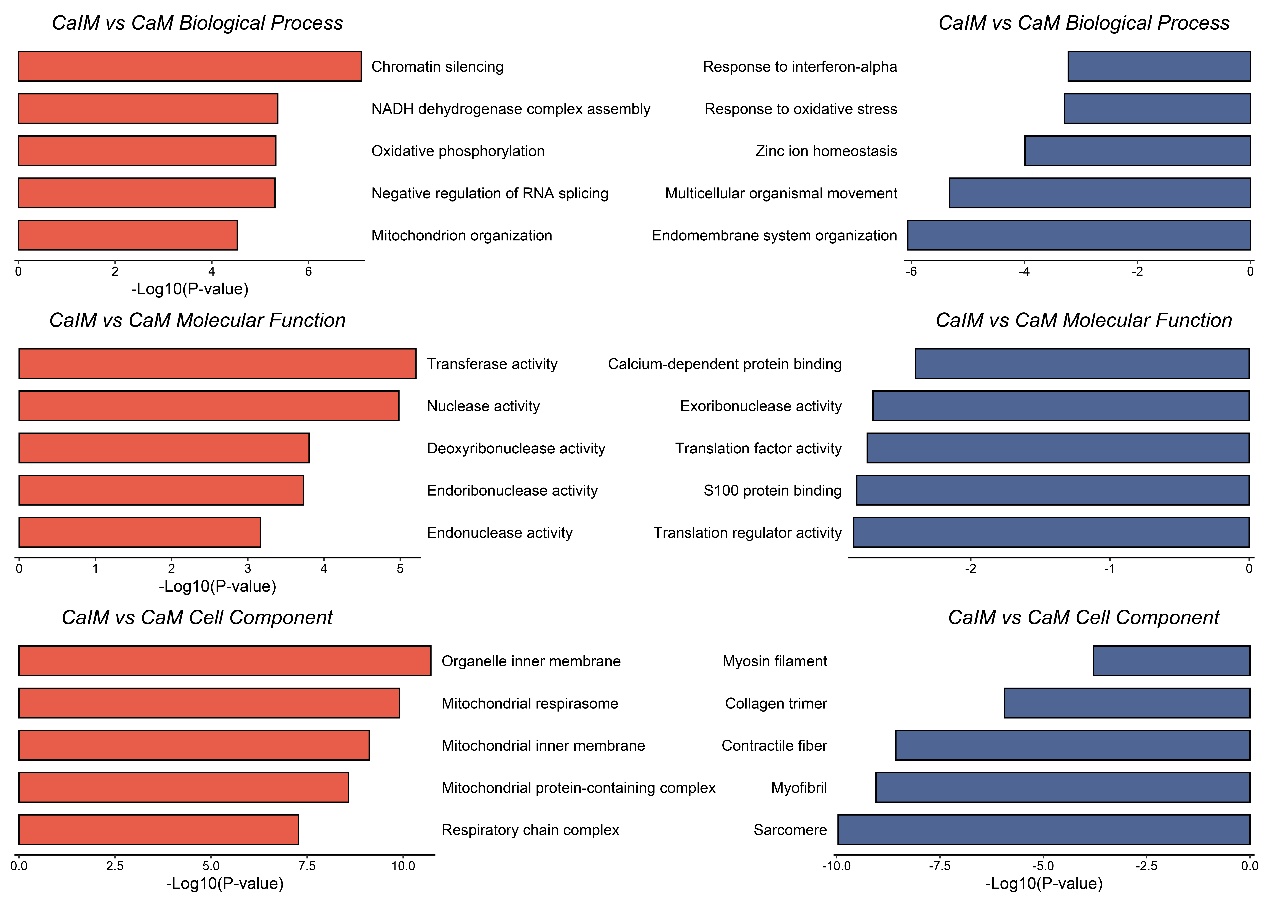


**Figure S25. GO enrichment analysis of CaIM versus CaM-regulated proteins.** GO enrichment analysis between CaIM and CaM groups in “Biological Process”, “Molecular Function”, and “Cellular Component” gene terms. Genes were enrolled using up-regulated or down-regulated proteins between CaIM and CaM groups.


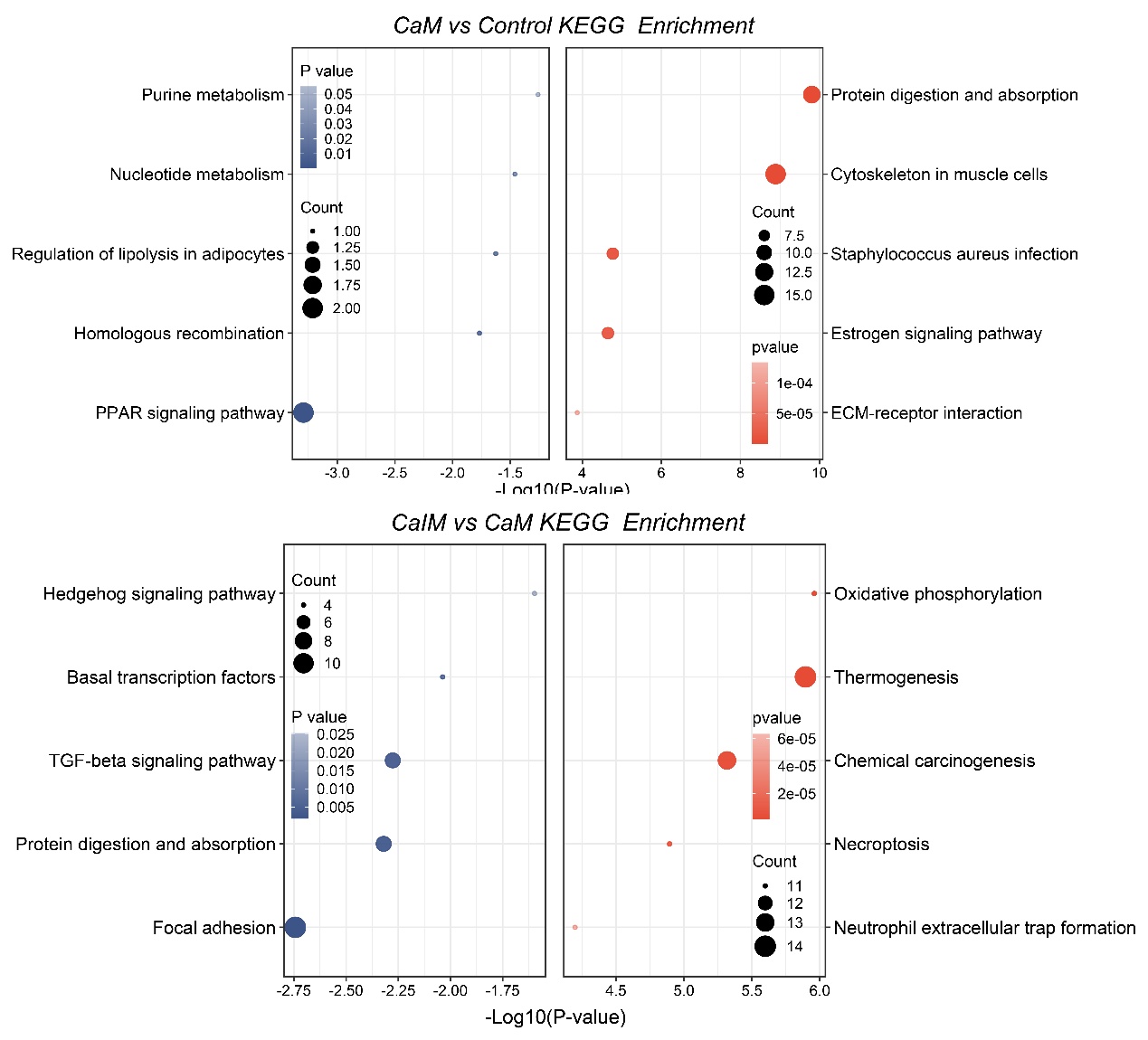


**Figure S26. KEGG pathway analysis of differentially expressed proteins.** KEGG enrichment analysis. KEGG pathways analysis of the differentially expressed proteins in CaM and control comparison (top) and CaIM and CaM comparison (bottom).


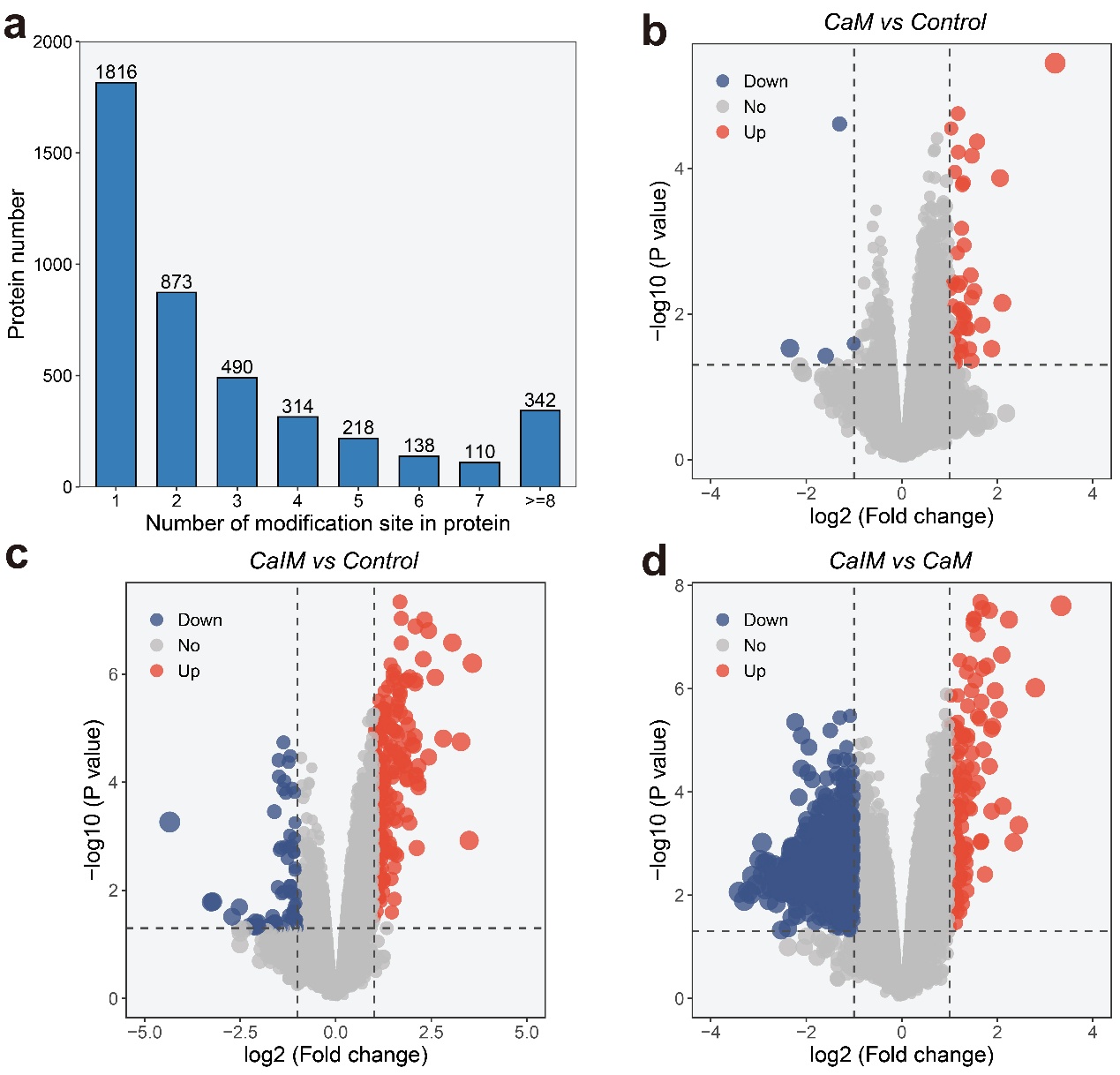


**Figure S27. Phosphoproteomic analysis of tumor tissues.** Phosphoproteomics analysis of tumor tissues after interventional therapy. a) Number of phosphorylation sites in phosphoprotein. b-d) Volcano plot showing differentially expressed phosphorylation sites in CaM and control comparison (b), CaIM and control comparison (c), CaIM and CaM comparison (d), respectively.


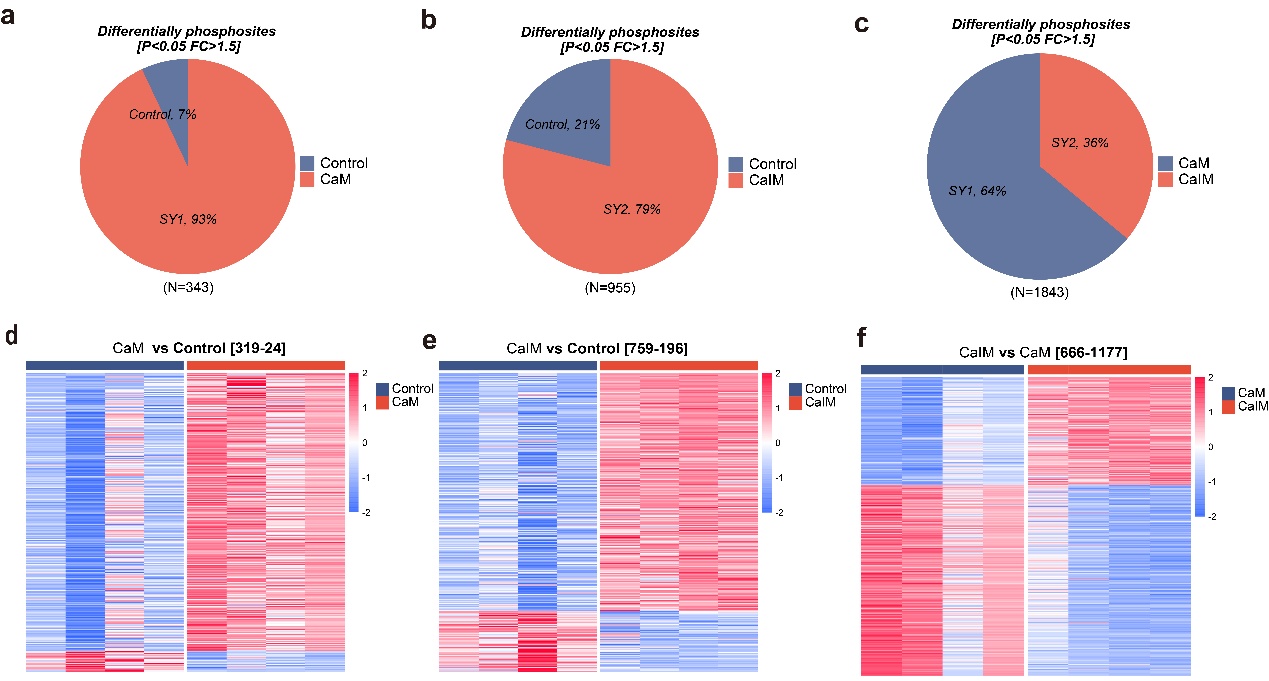


**Figure S28. Phosphoproteomic profiling across treatment groups.** Differential phosphorylation sites distribution among treatment groups based on phosphoproteomics data. a-c) Pie chart showing the up-regulated or down-regulated phosphorylation sites in CaM and control groups (a), CaIM and control groups (b), and CaIM and CaM groups (c), respectively. d-f) Heatmap of the up-regulated or down-regulated phosphorylation sites in CaM and control groups (d), CaIM and control groups (e), and CaIM and CaM groups (f), respectively.


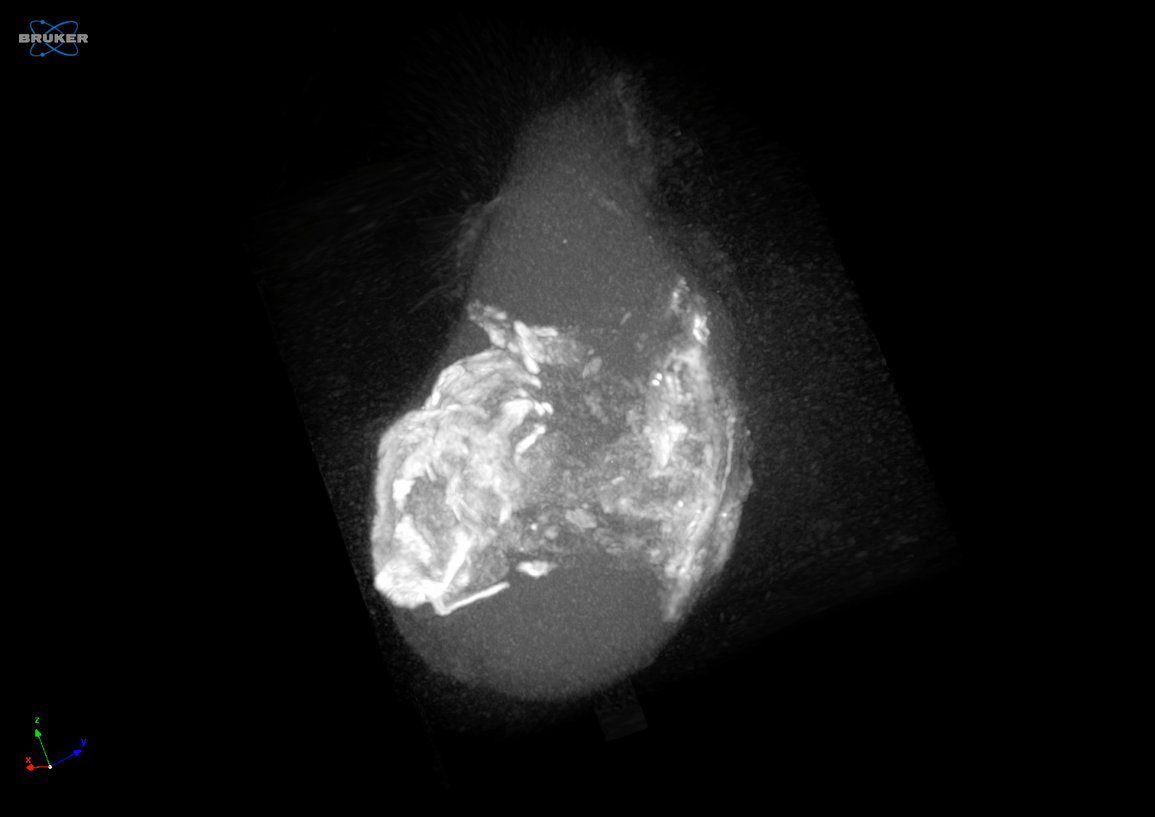
**Supporting Video**

**Video S1.** Rotational CT imaging of tumor calcification. Representative 0°-360° rotational CT of a CaIM-treated tumor showing continuous calcified regions and the spatial distribution of calcium deposition within the lesion. The video is available in the online repository: https://github.com/zzuliul/Rotational_CT.
